# The Indirect Effects of a Mindfulness Mobile App on Productivity Through Changes in Sleep Among Retail Employees: Secondary Analysis

**DOI:** 10.2196/40500

**Published:** 2022-09-28

**Authors:** Hallie Espel-Huynh, Matthew Baldwin, Megan Puzia, Jennifer Huberty

**Affiliations:** 1 Calm.com, Inc San Francisco, CA United States; 2 Behavioral Research and Analytics, LLC Salt Lake City, UT United States; 3 College of Health Solutions Arizona State University Phoenix, AZ United States

**Keywords:** mindfulness, mobile apps, workforce, workplace, sleep, presenteeism, mobile phone

## Abstract

**Background:**

Chronic sleep disturbance is prevalent among United States employees and associated with costly productivity impairment. Mindfulness interventions improve sleep (ie, insomnia and daytime sleepiness) and productivity outcomes, and mobile apps provide scalable means of intervention delivery. However, few studies have examined the effects of mindfulness mobile apps on employees, and no research to date has tested the role of sleep improvement as a potential mechanism of action for productivity outcomes.

**Objective:**

This study examined the effects of Calm, a consumer-based mindfulness app, and sleep coaching, on productivity impairment among retail employees through the indirect effects of changes in insomnia and daytime sleepiness.

**Methods:**

This study was a secondary analysis of data from a randomized controlled trial (N=1029) comparing the use of Calm (n=585, 56.9%) to a waitlist control (n=444, 43.2%) for 8 weeks among employees of a large retail employer in the United States. A subset of individuals with elevated insomnia symptoms also had access to brief sleep coaching with Calm (n=101, 9.8%). Insomnia symptom severity, daytime sleepiness, and productivity impairment (ie, absenteeism, presenteeism, overall productivity impairment, and non–work activity impairment) were assessed at baseline and weeks 2, 4, 6, and 8. Indirect effects were evaluated with latent growth curve modeling to test whether the Calm intervention (Calm group vs waitlist control) was effective in reducing work productivity impairment through changes in sleep disturbance.

**Results:**

No significant main effects of Calm intervention on productivity impairment were detected for any outcome at α level of .05, with the exception of non–work activity impairment models, in which Calm intervention reduced non–work activity impairment over time (*P*=.01 and *P*=.02 for insomnia and sleepiness models, respectively). Significant indirect effects of insomnia were detected for presenteeism (*P*=.002), overall work productivity (*P*=.01), and non–work activity impairment (*P*=.002); Calm intervention produced significantly greater reductions in insomnia symptoms (relative to waitlist control), and decreases in insomnia were associated with decreases in work productivity impairment. There was no significant indirect effect of change in insomnia on changes in absenteeism (*P*=.20). Furthermore, we detected no significant indirect effects of daytime sleepiness on productivity impairment.

**Conclusions:**

We found that Calm (plus sleep coaching for a small subset of individuals) had beneficial effects on employee sleep, and these benefits on sleep were related to indirect effects on productivity impairment (ie, presenteeism, overall work productivity impairment, and non–work activity impairment). There were no overall main effects of Calm intervention on productivity impairment; however, insomnia appears to be a mechanism associated with benefits for employee productivity. This is one of the first studies to suggest that sleep benefits of a mindfulness mobile app may also indirectly relate to benefits for workplace productivity.

**Trial Registration:**

ClinicalTrials.gov NCT05120310; https://clinicaltrials.gov/ct2/show/NCT05120310

## Introduction

### The Problem of Sleep Disturbance

Chronic sleep disturbance is widespread in the United States, with an estimated 36.3% of adults reporting at least one dimension of poor sleep [1]. Insomnia (characterized by difficulty falling asleep, staying asleep, or waking early, along with associated distress or impairment) is a common cause of sleep disturbance linked to substantial public health burden, including increased medical costs, poor mental health, and psychosocial impairment [2,3]. Sleep disturbances such as insomnia also contribute to the daytime effects of poor sleep quality [4], and an estimated one-third of individuals with insomnia experience excessive daytime sleepiness [5]. Insomnia and daytime sleepiness are particularly prevalent among the employed population, with an estimated 19.2% of all workers in the United States experiencing poor sleep quality [6], 23.2% reporting symptoms consistent with insomnia [7], and 16.2% of daytime workers experiencing excessive daytime sleepiness [6]. This paper seeks to summarize a gap in existing research on digital health solutions to address employee sleep and productivity concerns and evaluate the indirect effects of a commercial mindfulness app on employee productivity through sleep improvements.

Both insomnia and daytime sleepiness contribute to costly declines in work productivity (ie, productivity impairment) [6-11]. Compared with their peers with no sleep disturbance, individuals with insomnia experience more absenteeism (missed time from work), presenteeism (time spent being nonproductive at work) [6,7,11], overall work productivity impairment (absenteeism and presenteeism combined), and non–work activity impairment (ie, impairment in functioning outside of work hours) [12]. Daytime sleepiness (because of poor sleep quality or inadequate sleep duration) is associated with increased rates of presenteeism, overall work productivity impairment, and non–work activity impairment [13,14]. Overall, it is estimated that the United States loses an equivalent of 1.23 million working days because of insufficient sleep, which corresponds to approximately 9.9 million working hours [11].

### Benefits of Mindfulness in the Workplace

Several studies [15] have demonstrated the beneficial effects of mindfulness-based workplace interventions on employee sleep outcomes, including improved sleep quality, reduced daytime dysfunction, and reduced fatigue [16-18]. Such interventions may serve as an ideal first-line intervention strategy in the workplace, potentially increasing employees’ access to help for sleep problems, particularly for those who would not otherwise have access to sleep support [19]. To date, most studies have evaluated the effects of face-to-face interventions, which are costly and complex to implement in workplace settings, may require engagement with highly trained clinicians, and can involve complex payment structures [17,18]. Mobile app interventions have the potential to drastically increase reach and impact [20-23].

There is growing evidence supporting the efficacy of mobile-delivered mindfulness apps on sleep outcomes [20,21]. Various mindfulness apps have been shown to improve sleep in different populations [21], including adults with symptoms of insomnia [24,25], women experiencing environmental stress [21], and parents experiencing pandemic-related stress [26]. Calm is a commercially available mobile mindfulness app that has demonstrated efficacy in improving symptoms of sleep disturbance [24,27]. In addition to Calm’s large library of content to support mindfulness practice (eg, guided mindfulness meditations and mindful movements), Calm also includes sleep content that is specifically designed to facilitate improved sleep, including guided sleep meditations, sleep stories for relaxation at bedtime, and sleep-focused music and soundscapes. In a cross-sectional survey of Calm subscribers who used the Calm sleep content, users reported that Calm helped them fall asleep, stay asleep, and get more restful sleep [27]. Moreover, among adults with sleep disturbance, results from a randomized controlled trial indicated that Calm reduced daytime sleepiness, as well as other sleep-related concerns (ie, cognitive and somatic presleep arousal and fatigue), relative to waitlist controls [24].

### Gaps in Existing Evidence

To date, evidence for the utility of mobile apps in improving sleep in the workplace is limited. Most workplace evaluations of mindfulness apps have focused solely on mental health outcomes such as anxiety, depression, and psychological well-being [28,29]. Notably, few studies have focused specifically on sleep, especially when examining large-scale pragmatic app implementations across multisite employers [28,29]. Given the widespread prevalence of sleep disturbance in the workforce and its costly impact on productivity, as well as the potential for mindfulness apps to provide a scalable solution for employers, this is a crucial area for further study.

Our team recently evaluated the outcomes of a large randomized controlled trial evaluating Calm’s effects on 2 dimensions of sleep disturbance—insomnia and daytime sleepiness (in addition to mental health, resilience, productivity impairment, and health care visits)—among employees at a large retailer in the United States. The results indicated a significant benefit of Calm on insomnia symptoms and daytime sleepiness over the 8-week study period. In a smaller subsample of study completers (but not in the overall sample), significant reductions in employee productivity impairment were observed when examining measures of presenteeism, overall work productivity impairment (absenteeism and presenteeism combined), and non–work activity impairment. No effects were observed for absenteeism in either the full sample or the completer subsample [30].

The extent to which reductions in productivity impairment may occur through reductions in sleep disturbance has yet to be determined. We are aware of the lack of studies examining this potential link. However, given the strong relationships between sleep and productivity outcomes in the workplace, it is plausible that mindfulness apps may work indirectly on productivity impairment through improvements in sleep. Indeed, our team’s earlier work showed that the difference in mental health outcomes observed in individuals who received Calm (plus sleep coaching for a small subset of individuals with elevated insomnia symptoms) relative to the waitlist control occurred through an indirect effect of improvements in sleep [31]. Specifically, the results showed an indirect effect of Calm intervention on improvements in depression and anxiety through cognitive and somatic presleep arousal [31]. Interestingly, in that study, no significant indirect effects were found for daytime sleepiness (insomnia symptoms were not directly measured).

### Aims of This Analysis

To date, few studies have examined the effects of a consumer-based mindfulness mobile app on productivity outcomes in the workplace; we are aware of no studies that have assessed whether an app may provide indirect benefits in reducing productivity impairment through the mechanism of reduced sleep disturbance. This study was a secondary analysis of data from the study by Huberty et al [30] to examine whether Calm worked indirectly on productivity impairment (ie, absenteeism, presenteeism, overall productivity impairment, and non–work activity impairment) through the mechanisms of reduced insomnia and daytime sleepiness among employees of a large retailer in the United States.

## Methods

### Ethics Approval

This study was approved by the institutional review board of Arizona State University (STUDY00014072). All participants completed an electronic informed consent form before participation. The retail employer partner provided input on the study design as it pertained to their interest in supporting employee sleep and well-being as a means of improving productivity and performance.

### Participants and Recruitment

The study procedures are described in detail in the study by Huberty et al [30]. Primary outcomes for the clinical trial included insomnia and daytime sleepiness; secondary outcomes included productivity, resilience, depression, anxiety, and stress, as well as sleep diary outcomes (as assessed among individuals offered sleep coaching; see the *Intervention* section for details). Participants were recruited from a large retailer across 1096 work site locations (eg, retail and corporate) distributed throughout the United States between August and December 2021. The number of potentially- eligible employees was approximately 74,000. Recruitment occurred via email invitations distributed by human resources staff and store leaders, as well as on site via flyers posted in workplace break rooms. Email materials and flyers included a QR code and website link that directed participants to a web-based eligibility survey and electronic informed consent form, if eligible. To maintain a pragmatic approach and resemble procedures used if the app were offered as an employee benefit through the retail employer, all recruitment communications were deployed by the employer and not by research personnel. Employees were eligible for the study if they (1) were current employees of the company, (2) were aged at least 18 years, (3) were able to read and understand English, (4) owned a smartphone, (5) were willing to download the Calm app, and (6) had not practiced meditation for >60 minutes per month in the past 6 months [32]. As Calm is a commercially available app with wide name recognizability, potential participants were blinded to Calm during the recruitment process and were informed that the study aimed to test the effects of a *health and wellness app*. After consenting to and completing the baseline self-report measures, participants in the Calm intervention condition received email instructions to download the Calm app at no cost to them.

Participant randomization (Calm intervention vs waitlist control) occurred at the site level (stratified by the number of employees per work site) to reduce possible contamination effects because of the condition among employees at the same work site.

### Intervention

Participants in the Calm intervention group were instructed to use Calm autonomously for 10 minutes per day during the 8-week intervention period. To maintain consistency with naturalistic use patterns, no direction was provided regarding the specific content to be used. Weekly reminders to use the app were sent via SMS text messages (to a number specified by the participants); however, no additional communication or incentives were provided for app engagement. The Calm app includes a variety of content types, including guided meditation and breathing exercises, sleep stories, relaxing music, and ambient soundscapes. In addition to the Calm app access, all participants had the option to complete an initial, synchronous, 20-minute *virtual concierge* session with a Calm coach to orient them to the app’s offerings and potential areas for behavior change.

A subsample of Calm intervention group participants with elevated insomnia symptoms (≥10 on the Insomnia Severity Index [ISI]; 307/1029, 29.83%; see the *Measures* section) were invited to opt in to receive up to 6 sleep coaching sessions with a trained Calm sleep coach (ie, master’s-level behavioral health training and/or coaching certification from an accredited coaching program). Sleep coaching sessions addressed behavioral principles for managing sleep disturbance, including establishing a regular pattern of sleep, engaging in sleep hygiene practices, sleep restriction (as appropriate for insomnia symptoms), practicing bedtime mindfulness, and improving the sleep environment. Participants in the waitlist group were informed that they would receive access to the health and wellness app after completing their final assessment at week 8 (waitlist participants with elevated insomnia symptoms were also invited to participate in sleep coaching alongside access to Calm).

### Measures

#### Overview

Participants were asked to complete self-report assessments via the Qualtrics electronic survey platform every 2 weeks during the study period (baseline and weeks 2, 4, 6, and 8). For each assessment, participants received an email with a link to complete the web-based survey. Participants who completed the final survey were entered into a raffle for 1 of 5 Calm *swag* prize bags (which included Calm-branded pencils, notepads, and books). No other incentives were provided for the completion of the measures. The assessments included in this secondary analysis are described in this section. Additional outcomes assessed in the full randomized controlled trial included depression, anxiety, perceived stress, resilience, number of medical care visits, and app use (Calm intervention group only).

#### Baseline Demographic Characteristics

Demographic and individual characteristics (16 items assessing personal characteristics such as race, ethnicity, work, family, and medical history) were collected at baseline.

#### Insomnia Symptoms

Insomnia symptoms were assessed among all participants using the ISI [33], a 7-item self-report questionnaire assessing insomnia symptoms (eg, difficulty falling and staying asleep) during the past 2 weeks and the distress and impairment associated with the symptoms. Items are rated on a 5-point Likert-type scale, and the total scale scores are obtained by summing the item ratings. A cutoff score of ≥15 indicates moderate to severe insomnia symptoms in the clinical range [33]. The ISI has demonstrated good internal consistency (Cronbach α=.74 in the validation sample), sensitivity to change, and convergence with both objectively measured sleep disturbance and clinician ratings [33].

#### Daytime Sleepiness

Daytime sleepiness symptoms were measured using the Epworth Sleepiness Scale [34], which includes 8 items assessing recent dozing behavior during routine daytime activities (eg, sitting and reading and conversations). Items are rated on a 4-point Likert scale from 0 (*would never doze*) to 3 (*high chance of dozing*). The total scores are obtained by summing the item ratings (range 0-24, with higher scores indicating greater sleepiness). The Epworth Sleepiness Scale has shown high internal consistency (Cronbach α=.7-.9 in varying populations), demonstrates convergent validity with objective measures of sleepiness and sleep disturbance (ie, sleep latency), and differentiates between clinical and nonclinical sleep populations [34,35].

#### Productivity Impairment

The Work Productivity and Activity Impairment Questionnaire–General Health measure [36] is a 6-item, well-validated self-report scale that measures general physical and mental health–related impairments in work and nonwork activities, as well as absenteeism and presenteeism. Respondents are asked about current employment, hours missed because of health problems and other reasons, hours worked, and the degree to which health affected productivity during work and in other nonwork activities in the past 7 days. The 4 outcomes generated from the scale are the percentage of work time missed because of health (absenteeism), percentage of impairment while working because of health (presenteeism), percentage of overall work impairment because of health (overall work impairment), and percentage of non–work activity impairment because of health (non–work activity impairment). The Work Productivity and Activity Impairment Questionnaire–General Health has demonstrated sensitivity to productivity impairment in individuals with insomnia [37] and daytime sleepiness [14,37].

### Data Analyses

#### Data Preparation and Sample Size

All analyses were conducted using RStudio (build 443; 2022.02.0; version 4.1.2). Participants in the Calm and waitlist control groups were compared in terms of demographic characteristics and baseline sleep- and productivity-related variable scores via 2-tailed independent-sample *t* tests and Pearson chi-square tests as appropriate. In the parent randomized controlled trial, power analyses were conducted using G*Power (version 3.0), yielding a minimum sample size of N=364 to detect a significant effect of Calm on primary outcomes. An initial target sample of N*=*500 was selected (250 per group), anticipating approximately 30% attrition.

#### Indirect Effects

**Figure 1:**
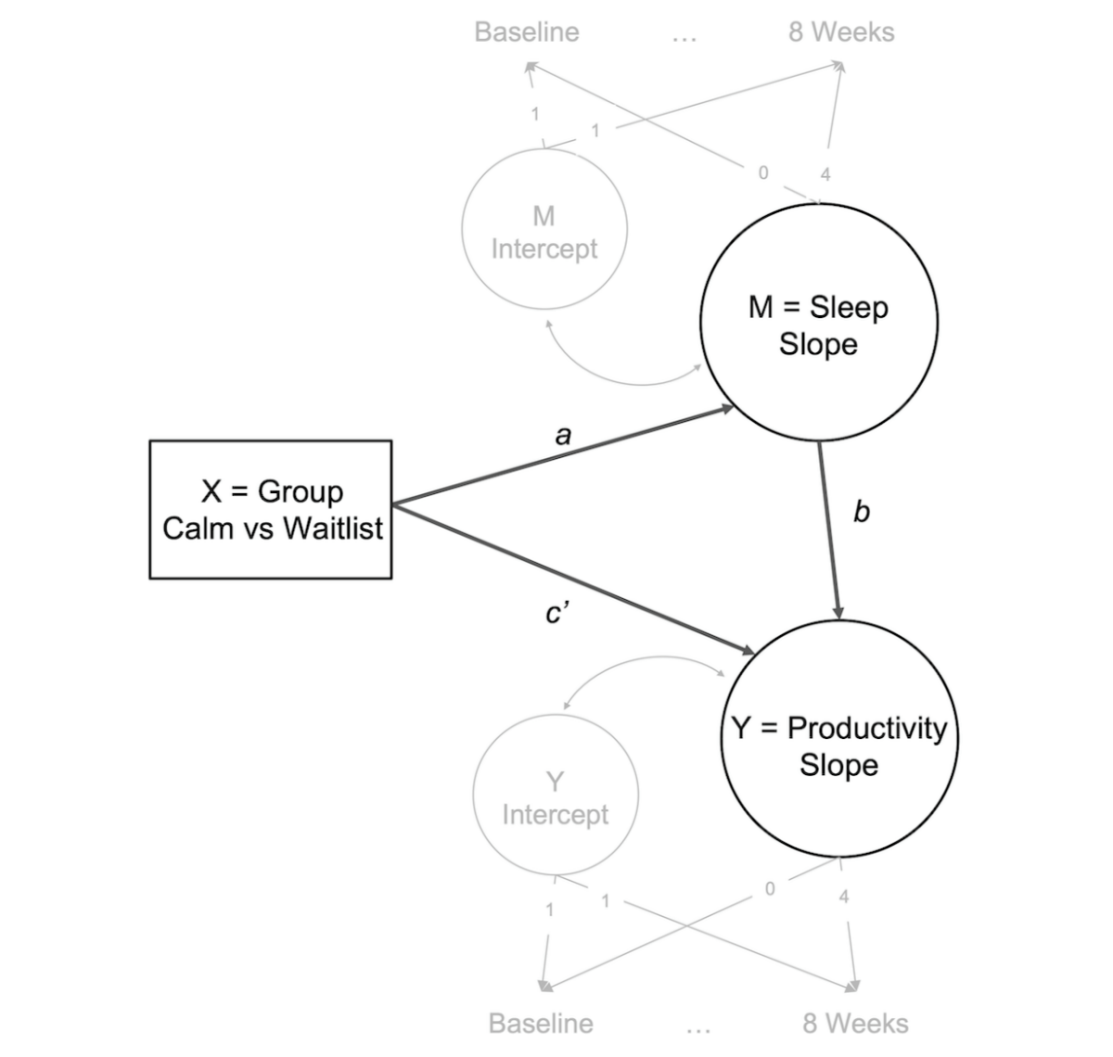
Conceptual depiction of the latent growth curve model, with primary elements of the indirect effect in bold. Mean structures, variances, and covariates were removed for simplicity. Factor loadings for baseline and 8 weeks are depicted, although all time points were used to estimate latent intercepts and slopes.

Indirect effects were evaluated using latent growth curve models [38] using the *lavaan* package in R [39]. We examined whether the Calm intervention (Calm group vs waitlist control) was effective in improving employee productivity outcomes (ie, latent change in absenteeism, presenteeism, overall productivity impairment, and non–work activity impairment from baseline to week 8) through indirect effects of changes in sleep disturbance (ie, latent change in insomnia and daytime sleepiness from baseline to week 8). Absenteeism percentages were zero-inflated and nonnormally distributed; thus, this variable was recoded as a dichotomous variable, indicating whether an individual reported any missed time during the 1-week before each assessment. Thus, mean level changes (ie, the latent slope) for absenteeism would reflect changes in the likelihood of being absent (vs not absent). All other outcomes were modeled continuously. Covariates were modeled as predictors of both mediator and outcome slopes and intercepts and included gender, hourly worker status, racial minority status, Hispanic ethnicity, presence of a sleep condition, presence of a chronic health condition, and presence of a mental health condition. Separate models were run for each mediator and outcome combination, and latent intercepts and slopes were estimated for both mediators and outcomes. Indicator residual variances were constrained to equality over time, and full information maximum likelihood estimation was used to account for missing data (Multimedia Appendices 1-3 show missing data reports). Regression paths were included for the effect of the intervention on the mediator (*a* path), mediator on the outcome (*b* path), and intervention on the outcome (*c′* path). The indirect effect was calculated as the product of the *a* and *b* paths [40,41] and was estimated using bias-accelerated and corrected bootstrapping (1000 resamples) [41]. A conceptual model of the analytical approach is shown in Figure 1. Consistent with recommendations for interpreting regression-based indirect effects models with bootstrapping, we did not require a significant total effect before exploration of indirect effects, as evidence suggests that a meaningful indirect effect can be present without the presence of a total effect, and bootstrapping does not rely on the *causal steps* approach used by traditional stepwise mediation methods [40,42].

## Results

### Sample Characteristics

The full trial included 1029 participants (n=585, 56.9% in the Calm group, and n=444, 43.2% in the waitlist control group), 192 (18.7%) of whom completed full assessments at all 5 time points. A description of participant demographics and other baseline characteristics was reported by Huberty et al [30]. Briefly, 50.6% (518/1024) of the participants identified as female, 25.3% (260/1029) identified as belonging to a racial minority group, and 15.5% (159/1029) identified as Hispanic. In terms of clinical characteristics and sleep concerns, 25.97% (267/1028) of the participants scored ≥15 on the ISI, indicating a likely diagnosis of clinical insomnia. Among those who screened positive for insomnia according to their ISI scores, only 32.2% (86/267) indicated that they had been diagnosed with insomnia. Of the included Calm participants, 63.4% (370/584) were screened as eligible for sleep coaching. Of those 370 participants, 101 (27.3%) opted in. In addition, 25.5% (217/851) endorsed the presence of ≥1 diagnosed sleep condition (eg, insomnia, sleep apnea, and restless leg syndrome), and 57.9% (574/992) of the sample indicated that they had been diagnosed with at least one chronic physical health condition (eg, diabetes, hypertension, and chronic asthma). The characteristics of all participants are described in Table 1.

The Calm and waitlist groups did not differ significantly at baseline in terms of gender, race, ethnicity, presence of a chronic medical condition, or presence of a diagnosed sleep condition (including insomnia; all *P*≥.13; Table 1). The waitlist group had a significantly higher proportion of hourly workers (vs salaried; *χ*^2^_1_=17.4; *P*<.001) and self-reporting of a diagnosed mental health condition (*χ*^2^_1_=5.5; *P*=.02). The groups did not differ significantly at baseline in insomnia symptom severity, daytime sleepiness, absenteeism, presenteeism, or overall work productivity impairment (Table 2); however, the waitlist group had significantly greater non–work activity impairment (*P*=.047). Multimedia Appendix 4 shows the group means across all time points. As reported by Huberty et al [30], only 15% (88/585) in the Calm group and 43.2% (192/444) in the waitlist control group provided data for all measures at all time points. In the primary outcome analysis, participants with complete assessment data (ie, all 5 time points) were more likely to identify with a racial minority group (ie, endorsed at least one race other than White), be non-Hispanic, and work on salaried income versus hourly wage [30]. Multimedia Appendices 1-3 show a complete missing data report.

**Table 1:** Demographic and baseline characteristics of the sample (N=1029).

Characteristic		Waitlist (n=444), n (%)	Calm (n=585), n (%)	Chi-square (*df*)^a^	*P* value
**Gender**					
	Man	202 (45.7)	272 (46.7)	0.1 (1)	.80
	Woman	226 (51.1)	292 (50.2)	0.1 (1)	.80
	Other	14 (3.2)	18 (3.1)	0.1 (1)	.80
**Race**					
	American Indian or Alaskan Native	12 (2.7)	16 (2.7)	0.0 (1)	.98
	Asian or Asian American	16 (3.6)	33 (5.7)	2.3 (1)	.13
	White or European American	340 (77.1)	463 (79.4)	0.8 (1)	.37
	Black or African American	26 (5.9)	30 (5.2)	0.3 (1)	.60
	Biracial or multiracial	27 (6.1)	26 (4.5)	1.4 (1)	.23
	Other	33 (7.5)	20 (3.4)	8.4 (1)	.004
**Ethnicity**					
	Not Hispanic or Latino	367 (82.7)	503 (86)	1.9 (1)	.17
	Hispanic or Latino	77 (17.3)	82 (14)	1.9 (1)	.17
**Employee type**					
	Salaried	141 (31.8)	262 (44.8)	17.4 (1)^b^	<.001^b^
	Hourly	303 (68.2)	323 (55.2)	17.4 (1)^b^	<.001^b^
**Insomnia screening (Insomnia Severity Index)**					
	Moderate or severe insomnia	148 (33.3)	173 (29.6)	1.4 (1)	.23
	Unlikely insomnia diagnosis	296 (66.7)	411 (70.4)	1.4 (1)	.23
**Chronic health conditions**					
	At least one	254 (57.2)	320 (56.3)	1.1 (1)	.29
	None	170 (38.3)	248 (43.7)	1.1 (1)	.29
**Mental health conditions**					
	At least one	175 (41.3)	193 (34)	5.2 (1)^b^	.02^b^
	None	249 (58.7)	375 (66)	5.2 (1)^b^	.02^b^
**Sleep-related conditions**					
	At least one	96 (26.6)	121 (24.7)	0.3 (1)	.58
	None	265 (73.4)	369 (75.3)	0.3 (1)	.58

^a^Consistent with operational definitions of demographic covariates in the models of outcomes over time, chi-square tests reflect group comparisons of proportions of male and female, White and racial minority, Hispanic and non-Hispanic, completed and not completed a college education, salaried and hourly employment status, proportions of the likelihood of having insomnia based on the Insomnia Severity Index, presence or absence of a chronic health condition, and the presence or absence of a sleep-related condition.

^b^Significant differences from group comparisons.

**Table 2:** Baseline scores on outcomes of interest by group (N=1029).

Measure^a^	Waitlist (n=444)		Calm (n=585)		*t* test (*df*)	*P* value
	Values, n (%)	Values, mean (SD)	Values, n (%)	Values, mean (SD)		
ISI^b^	444 (100)	11.90 (5.87)	584 (99.8)	11.45 (5.65)	1.22 (1027)	.22
ESS^c^	443 (99.8)	7.10 (4.61)	582 (99.5)	7.26 (4.92)	0.51 (1024)	.61
WPAI^d^: absenteeism	415 (93.5)	0.23 (0.42)	559 (95.6)	0.19 (0.39)	1.77 (973)	.18
WPAI: presenteeism	406 (91.4)	30.62 (26.14)	547 (93.5)	28.10 (25.69)	1.48 (953)	.14
WPAI: overall work impairment	404 (91)	33.08 (28.44)	546 (93.3)	30.30 (27.93)	1.50 (949)	.13
WPAI: activity impairment	438 (98.6)	35.87 (28.70)	574 (98.1)	32.33 (27.16)	1.99^e^ (1011)	.047^e^

^a^All measures were assessed continuously, except for absenteeism, which was coded as a dichotomous variable (0=any health-related absence and 1=no absence).

^b^ISI: Insomnia Severity Index.

^c^ESS: Epworth Sleepiness Scale.

^d^WPAI: Work Productivity and Activity Impairment.

^e^Significant differences from group comparisons.

### Indirect Effects Analysis Results

The parameter estimates and significance tests for all growth curve models can be found in Multimedia Appendix 5.

#### Sleep Disturbance and Absenteeism

The model testing the indirect effects of Calm on absenteeism outcomes through insomnia over time showed a good fit. We found a significant beneficial effect of the Calm intervention on changes in insomnia (*a* path) but no significant effect of change in insomnia on change in absenteeism (*b* path). There was no significant direct effect of the Calm intervention on changes in absenteeism, accounting for changes in insomnia (*c*′ path), nor was there a significant indirect effect of change in insomnia (*a×b* path).

The model testing the indirect effects of Calm on absenteeism outcomes through daytime sleepiness over time also showed a good fit. We found a significant effect of the Calm intervention on changes in daytime sleepiness (*a* path) but no significant effect of change in daytime sleepiness on change in absenteeism (*b* path). There was no significant direct effect of the Calm intervention on absenteeism, accounting for the effects of daytime sleepiness (*c*′ path), nor was there a significant indirect effect of change in daytime sleepiness (*a×b* path). The model fit and coefficient estimates for both absenteeism models are presented in Table 3.

**Table 3:** Estimates for models evaluating the indirect effects of Calm on absenteeism through sleep disturbance.

Parameters		Model estimates			
		*b* (SE)	β	*P* values	95% CI
**Insomnia symptoms^a^**					
	Effect of Calm on Δ insomnia (*a* path; *X*→*M*)	−0.560^b^ (0.128^b^)	−0.257^b^	<.001^b^	−0.825 to −0.302^b^
	Effect of Δ in insomnia on Δ in absenteeism (*b* path; *M→Y*)	0.009 (0.006)	.317	.16	−0.003 to 0.021
	Total effect of Calm on Δ in absenteeism (*c* path; sum of direct and indirect effects)	0.002 (0.009)	.038	.80	−0.015 to 0.021
	Direct effect of Calm on Δ in absenteeism (*c′* path; *X→Y,* accounting for *M*)	0.007 (0.010)	.120	.48	−0.012 to 0.029
	Indirect effect of Δ in insomnia (*a×b*)	−0.005 (0.004)	−0.081	.20	−0.015 to 0.001
**Daytime sleepiness^c^**					
	Effect of Calm on Δ in daytime sleepiness (*a* path; *X→M*)	−0.261^b^ (0.092^b^)	−0.198^b^	.01^b^	−0.468 to −0.091^b^
	Effect of Δ in daytime sleepiness on Δ in absenteeism (*b* path; *M→Y*)	−0.011 (0.019)	−0.240	.56	−0.043 to 0.012
	Total effect of Calm on Δ in absenteeism (*c* path; sum of direct and indirect effects)	0.002 (0.009)	.032	.82	−0.016 to 0.020
	Direct effect of Calm on Δ in absenteeism (*c′* path; *X→Y,* accounting for *M*)	−0.001 (0.010)	.929	.93	−0.015 to 0.017
	Indirect effect of Δ in daytime sleepiness (*a×b*)	0.003 (0.005)	.047	.60	−0.003 to 0.014

^a^Root mean square error of approximation 0.023, comparative fit index 0.976, and Tucker-Lewis index 0.971.

^b^Significant differences from group comparisons.

^c^Root mean square error of approximation 0.032, comparative fit index 0.952, and Tucker-Lewis index 0.942.

#### Sleep Disturbance and Presenteeism

The model testing the indirect effects of Calm on presenteeism outcomes through insomnia over time showed an acceptable fit. We found a significant beneficial effect of the Calm intervention on changes in insomnia (*a* path) and a significant effect of change in insomnia on change in presenteeism (*b* path), such that greater reductions in insomnia symptoms were associated with greater reductions in presenteeism (ie, improved productivity). There was no significant direct effect of the Calm intervention on presenteeism, accounting for the effects of insomnia (*c*′ path); however, a significant indirect effect of change in insomnia (*a×b* path) was detected. Thus, Calm decreased insomnia, and decreases in insomnia were associated with increases in productivity (decreased presenteeism).

The model testing the indirect effects of Calm on presenteeism through daytime sleepiness over time showed a good fit. We found a significant effect of the Calm intervention on changes in daytime sleepiness (*a* path) but no significant effect of change in daytime sleepiness on change in presenteeism (*b* path). There was no significant direct effect of Calm on presenteeism, accounting for the effects of daytime sleepiness (*c*′ path) and no significant indirect effect of change in daytime sleepiness (*a×b* path). Table 4 presents the model fit and coefficient estimates for both presenteeism models.

**Table 4:** Estimates for models evaluating the indirect effects of Calm on presenteeism through sleep disturbance.

Parameter			Model estimates						
			*b* (SE)		β		*P* value		95% CI
**Insomnia symptoms^a^**									
	Effect of Calm on Δ in insomnia (*a* path; *X→M*)	−0.553^b^ (0.125^b^)		−0.258^b^		<.001^b^		−0.798 to −0.321^b^	
	Effect of Δ in insomnia on Δ in presenteeism (*b* path; *M→Y*)	3.236^b^ (0.798^b^)		.821^b^		<.001^b^		2.345 to 4.518^b^	
	Total effect of Calm on Δ in presenteeism (*c* path; sum of direct and indirect effects)	−0.943 (0.670)		−0.112		.16		−2.368 to 0.329	
	Direct effect of Calm on Δ in presenteeism (*c′* path; *X→Y,* accounting for *M*)	0.848 (0.740)		.100		.25		−0.547 to 2.215	
	Indirect effect of Δ in insomnia (*a×b*)	−1.791^b^ (0.579^b^)		−0.212^b^		.002^b^		−2.867 to −0.926^b^	
**Daytime sleepiness^c^**									
	Effect of Calm on Δ in daytime sleepiness (*a* path; *X→M*)	−0.257^b^ (0.094^b^)		−0.194^b^		.006^b^		−0.458 to −0.073^b^	
	Effect of Δ in daytime sleepiness on Δ in presenteeism (*b* path; *M→Y*)	1.103 (6.140)		.175		.86		−0.937 to 3.482	
	Total effect of Calm on Δ in presenteeism (*c* path; sum of direct and indirect effects)	−0.898 (0.675)		−0.108		.18		−2.117 to 0.597	
	Direct effect of Calm on Δ in presenteeism (*c′* path; *X→Y*, accounting for *M*)	−0.615 (1.837)		−0.074		.74		−1.940 to 1.135	
	Indirect effect of Δ in daytime sleepiness (*a×b*)	−0.284 (1.719)		−0.034		.87		−1.466 to 0.146	

^a^Root mean square error of approximation 0.053, comparative fit index 0.911, and Tucker-Lewis index 0.891.

^b^Significant differences from group comparisons.

^c^Root mean square error of approximation 0.038, comparative fit index 0.948, and Tucker-Lewis index 0.937.

#### Sleep Disturbance and Overall Work Productivity Impairment

The model testing the indirect effect of Calm on overall work impairment outcomes through insomnia over time showed a good fit. We found a significant beneficial effect of the Calm intervention on changes in insomnia (*a* path) and a significant effect of change in insomnia on change in presenteeism (*b* path), such that greater reductions in insomnia were predictive of greater reductions in productivity impairment. There was no significant direct effect of the Calm intervention on overall work impairment, accounting for the effects of insomnia (*c*′ path); however, a significant indirect effect of change in insomnia (*a×b* path) was detected. Thus, Calm decreased insomnia, and decreases in insomnia were associated with decreases in productivity impairment.

The model testing the indirect effect of Calm on overall work impairment outcomes through daytime sleepiness over time showed a good fit. We found a significant effect of the Calm intervention on changes in daytime sleepiness (*a* path) but no significant effect of change in daytime sleepiness on change in work impairment (*b* path). There was no significant direct effect of the Calm intervention on work impairment, accounting for the effects of daytime sleepiness (*c*′ path), and no significant indirect (mediating) effect of change in daytime sleepiness (*a×b* path). The model fit and coefficient estimates for both models are presented in Table 5.

**Table 5:** Estimates for models evaluating the indirect effects of Calm on work productivity impairment outcomes through sleep disturbance.

Parameter		Model estimates			
		*b* (SE)	β	*P* value	95% CI
**Insomnia symptoms^a^**					
	Effect of Calm on Δ in insomnia (*a* path; *X→M*)	−0.552^b^ (0.127^b^)	−0.257^b^	<.001^b^	−0.801 to −0.301^b^
	Effect of Δ in insomnia on Δ in overall work impairment (*b* path; *M→Y*)	3.129^b^ (0.893^b^)	.825^b^	<.001^b^	2.185 to 4.534^b^
	Total effect of Calm on Δ in overall work impairment (*c* path; sum of direct and indirect effects)	−1.018 (0.693)	−0.125	.14	−2.380 to 0.320
	Direct effect of Calm on Δ in overall work impairment (*c′* path; *X→Y*, accounting for *M*)	0.708 (0.855)	.087	.40	−0.700 to 2.203
	Indirect effect of Δ in insomnia (*a×b*)	−1.726^b^ (0.684^b^)	−0.212^b^	.01^b^	−2.851 to −0.898^b^
**Daytime sleepiness^c^**					
	Effect of Calm on Δ in daytime sleepiness (*a* path; *X→M*)	−0.257^b^ (0.095^b^)	−0.194^b^	.007^b^	−0.449 to −0.082^b^
	Effect of Δ in daytime sleepiness on Δ in overall work impairment (*b* path; *M→Y*)	0.978 (9.764)	.162	.92	−0.996 to 3.612
	Total effect of Calm on Δ in overall work impairment (*c* path; sum of direct and indirect effects)	−0.971 (0.690)	−0.121	.16	−2.284 to 0.431
	Direct effect of Calm on Δ in overall work impairment (*c′* path; *X→Y*, accounting for *M*)	−0.720 (2.295)	−0.090	.75	−2.175 to 1.022
	Indirect effect of Δ in daytime sleepiness (*a×b*)	−0.251 (2.164)	−0.031	.91	−3.648 to 0.163

^a^Root mean square error of approximation 0.049, comparative fit index 0.921, and Tucker-Lewis index 0.904.

^b^Significant differences from group comparisons.

^c^Root mean square error of approximation 0.039, comparative fit index 0.947, and Tucker-Lewis index 0.935.

#### Sleep Disturbance and Non–Work Activity Impairment

The model testing the indirect effect of Calm on non–work activity impairment outcomes through insomnia over time showed an acceptable fit. We found a significant beneficial effect of the Calm intervention on changes in insomnia (*a* path) and a significant effect of change in insomnia on change in activity impairment (*b* path), such that greater improvements in insomnia also corresponded to greater reductions in non–work activity impairment (ie, improved functioning). There was no significant direct effect of the Calm intervention on non–work activity impairment, accounting for the effects of insomnia (*c*′ path), although the total effect was significant. A significant indirect effect of change in insomnia (*a×b* path) was detected. Thus, Calm decreased insomnia, and decreases in insomnia were associated with decreases in non–work activity impairment.

The model testing the indirect effect of Calm on non–work activity impairment outcomes through daytime sleepiness over time showed a good fit. We found a significant effect of the Calm intervention on changes in daytime sleepiness (*a* path) but no significant effect of change in daytime sleepiness on change in activity impairment (*b* path). The Calm intervention had a significant direct effect on activity impairment (*c* path); however, this effect was not significantly mediated by an indirect effect of the change in daytime sleepiness (*a×b* path). The model fit and coefficient estimates for both models are presented in Table 6.

**Table 6:** Estimates for models evaluating sleep disturbance as a mediator of non–work activity impairment outcomes.

Parameter			Model estimates						
			*b* (SE)		β		*P* value		95% CI
**Insomnia symptoms^a^**									
	Effect of Calm on Δ in insomnia (*a* path; *X→M*)	−0.553^b^ (0.129^b^)		−0.257^b^		<.001^b^		−0.822 to−0.306^b^	
	Effect of Δ in insomnia on Δ in non–work activity impairment (*b* path; *M→Y*)	3.173^b^ (0.705^b^)		.736^b^		<.001^b^		2.050 to 4.281^b^	
	Total effect of Calm on Δ in non–work activity impairment (*c* path; sum of direct and indirect effects)	−1.647^b^ (0.673^b^)		−0.178^b^		.01^b^		−2.933 to −0.176^b^	
	Direct effect of Calm on Δ in non–work activity impairment (*c′* path; *X→Y,* accounting for *M*)	0.109 (0.754)		.012		.89		−1.360 to 1.521	
	Indirect effect of Δ in insomnia (*a×b*)	−1.756^b^ (0.565^b^)		−0.384^b^		.002^b^		−2.995 to −0.871^b^	
**Daytime sleepiness^c^**									
	Effect of Calm on Δ in daytime sleepiness (*a* path; *X→M*)	−0.257^b^ (0.094^b^)		−0.194^b^		.006^b^		−0.456 to −0.072^b^	
	Effect of Δ in daytime sleepiness on Δ in non–work activity impairment (*b* path; *M→Y*)	1.345 (6.757)		.195		.84		−0.761 to 3.809	
	Total effect of Calm on Δ in nonwork activity impairment (*c* path; sum of direct and indirect effects)	−1.609^b^ (0.691^b^)		−0.176^b^		.02^b^		−2.917 to −0.195^b^	
	Direct effect of Calm on Δ in non–work activity impairment (*c′* path; *X→Y,* accounting for *M*)	−1.263 (2.114)		−0.138		.55		−2.626 to 0.446	
	Indirect effect of Δ in daytime sleepiness (*a×b*)	−0.346 (2.025)		−0.038		.87		−1.374 to 0.135	

^a^Root mean square error of approximation 0.058, comparative fit index 0.906, and Tucker-Lewis index 0.885.

^b^Significant differences from group comparisons.

^c^Root mean square error of approximation 0.039, comparative fit index 0.952, and Tucker-Lewis index 0.941.

## Discussion

### Principal Findings

The primary aim of this study was to examine whether the beneficial effects of a Calm mobile app intervention for sleep problems (namely, insomnia and daytime sleepiness) would result in indirect benefits for productivity (ie, absenteeism, presenteeism, overall productivity impairment, and non–work activity impairment) among employees of a large retailer in the United States. Web-based, synchronous sleep coaching was also provided for a small subset of those with elevated insomnia symptoms). Even in the absence of significant main effects of the complete Calm intervention on productivity outcomes, we found that changes in insomnia indirectly influenced the effects of Calm intervention on presenteeism, overall productivity impairment, and non–work activity impairment over the 8-week study period. For all 3 outcomes, Calm intervention exerted indirect effects on productivity impairment by producing significant reductions in insomnia symptoms over time, which corresponded to greater reductions in productivity impairment over time. There were no significant indirect effects of Calm intervention on absenteeism through insomnia or on any of the 4 productivity impairment outcomes through daytime sleepiness. Therefore, although the Calm intervention was effective in reducing both insomnia symptoms and daytime sleepiness, it would likely be the reductions in insomnia symptoms that would have primary benefits for productivity. This is one of the first studies to suggest that a mindfulness meditation mobile app implemented in the workplace may reduce productivity impairment through the mechanism of improved sleep. Additional research is needed to replicate and confirm these findings using other employee samples. Although the fit statistics suggested that our models provided an acceptable to good fit for the data, replication studies should also explore additional employee retention strategies (eg, incentives for assessment completion) to increase data availability and enhance confidence in our model estimates. Furthermore, future work could focus more specifically on causal relationships between engagement with the app or coaching, sleep, and work productivity over time.

### Changes in Insomnia Symptoms as a Mediator of Productivity Outcomes

It is not surprising that a mindfulness mobile app intervention may improve productivity by reducing insomnia symptoms over time. This finding is consistent with the results from a recent randomized controlled trial, in which sleep-disturbed adults who used Calm experienced reductions in cognitive and somatic presleep arousal, which are 2 aspects of sleep that are closely linked to insomnia [24]. A follow-up analysis from that study showed that improvements in presleep arousal (but not daytime sleepiness) also served as a mechanism of action [31] by which Calm improved other dimensions of well-being (ie, anxiety and depression). Cognitive and somatic presleep arousal refers to the inability to *shut off* one’s thoughts (cognitive) and relax physically (somatic) in preparation for sleep. Both are elevated among individuals with insomnia and are closely related to the core symptoms of insomnia disorder [3,43,44]. Notably, approximately one-fourth of the participants screened positive for a likely insomnia diagnosis with at least moderate clinical symptoms, and only approximately one-third of those individuals self-reported the same diagnosis. This suggests that many employees may not be aware of and/or have access to care for their clinical insomnia symptoms. Without tools such as mindfulness apps to address their symptoms, this illustrates a missed opportunity to influence employee sleep and productivity.

We also did not find a significant indirect effect of the Calm intervention on absenteeism through changes in insomnia symptoms. In the available literature, absenteeism has been less consistently related to sleep disturbance in the workplace than to other measures of productivity impairment [7]. We also observed relatively low rates of health-related absenteeism at baseline (ie, only 204/974, 20.9%, of the sample reported an absence at baseline). Thus, the effects of the intervention on absenteeism may be relevant for only a small minority of retail employees.

Employers incur immense costs because of insomnia-related productivity impairments. Our findings suggest that reductions in insomnia symptoms may be a key driver of reduced productivity impairment for employees and that mindfulness apps such as Calm, plus additional coaching support where indicated, can serve as accessible and scalable first-line interventions to address both. Future work could examine how specific content—–or use at particular times of day—may affect sleep and productivity. For example, one could examine whether it is more beneficial to use sleep content at bedtime, general mindfulness meditation while winding down in the evening, or a combination of both, as well as whether this varies by employee or work schedule (eg, varying shift work vs consistent schedule). Furthermore, one may examine whether coaching works synergistically with the app itself, such that app use increases after certain coaching interactions.

### Change in Daytime Sleepiness as a Mediator of Productivity Outcomes

It is somewhat surprising that the Calm intervention did not appear to work indirectly on productivity impairment through changes in daytime sleepiness, especially given that daytime sleepiness is closely linked to workplace productivity and manifests primarily during work hours. However, this finding is consistent with results from a prior randomized controlled trial evaluating the effects of Calm in adults with sleep disturbance. In that study, Calm reduced symptoms of daytime sleepiness, and these changes were directly associated with (but did not mediate) reductions in anxiety and depression [24]. Daytime sleepiness is also driven by many other sleep concerns, in addition to insomnia and insufficient sleep duration, including medical problems such as obstructive sleep apnea, which are not likely to be improved by meditation [45]. Thus, although commercial mindfulness apps may improve daytime sleepiness in certain cases, this may be a secondary benefit rather than a mechanism of action.

Therefore, the primary benefit of a mindfulness mobile app intervention on productivity impairment appears to be in helping users fall asleep more easily and reduce their distress about sleep (which is a key feature of insomnia), which presumably improves their ability to focus and be productive at work. Mechanistically, mindfulness practice encourages nonattachment to one’s internal experience (eg, racing thoughts, tense body, and difficulty settling for bedtime) [46], which may be particularly beneficial when attempting to fall asleep or go back to sleep after nighttime awakening. Taken together, these findings suggest that addressing insomnia symptoms is key to reducing work productivity impairment and non–work activity impairment among retail employees.

### Strengths

This study builds on existing knowledge about the benefits of implementing a mindfulness meditation mobile app in a workplace setting and its ability to affect productivity impairment through improvements in sleep. The strengths of this study include (1) a pragmatic randomized trial in a diverse sample of employees of a large retail company with sites distributed across the United States, thus maximizing generalizability to other US workplace settings; (2) a mechanistic approach to enhance our knowledge about *how* mobile mindfulness apps such as Calm, along with supplemental coaching, may reduce productivity impairment in employees; (3) frequent assessment of outcomes (ie, every 2 weeks throughout the study period), which allowed for a more rigorous evaluation of indirect effects; and (4) naturalistic use among participants (ie, participants were instructed to use the app for 10 minutes per day but received no other instructions or curation of content). Thus, employee engagement with Calm (and the observed benefit) was likely representative of how employees would use the app and associated services if they were offered as an employee benefit.

### Limitations

Despite its strengths, this study had several limitations. First, there was a large degree of attrition, leading to substantial missingness and potential bias in our parameter estimates (see Multimedia Appendices 1-3 for missing data reports), which may also correspond to a more limited engagement with the Calm intervention. There were multiple barriers to communicating with employees throughout the recruitment and study periods, which contributed to attrition (reported in the study by Huberty et al [30]). We attempted to account for any systematic differences between included and nonincluded cases by including covariates for any demographic characteristics that could be related to differential study attrition and patterns of missingness among outcome variables and covariates. Although data were not missing completely at random, we considered that data could be assumed to be missing at random and that our selected covariates could explain some of the missingness of key outcomes. The full information likelihood estimator, alongside the inclusion of strong auxiliary variables, has been shown to reproduce unbiased estimates of effects, even when missingness is initially nonignorable [47,48]. Considering that participants in this study shared a large degree of initial data (eg, demographics and baseline measures of sleep and productivity outcomes) and that included covariates were related to missingness, we feel confident that the full information and auxiliary variable approach we used here adequately accounts for missing data. Future studies with more comprehensive implementation strategies are required to overcome some of the barriers encountered with employee communication, participation, and attrition. Second, although participants were diverse in terms of gender, wage type, and the types of experienced medical and mental health concerns, the study sample was limited in terms of racial and ethnic diversity. Thus, future studies with more focused recruitment efforts on employees from diverse racial and ethnic backgrounds are warranted. Third, this study involved the Calm app plus sleep coaching, and it is unclear which effects can be attributed to the Calm app alone versus sleep coaching intervention. Finally, this study is limited by its use of self-report measures, with no formal clinician assessment of sleep disturbances. Future work may incorporate more formal sleep assessment data, for example, by using objective monitoring (ie, actigraphy), direct clinician observation, or the incorporation of employee data from medical claims or electronic health records.

### Conclusions

Commercial mindfulness apps provide a unique opportunity to deploy easily accessible and scalable interventions that can improve employees’ sleep and productivity. Mindfulness interventions are increasingly being shown to improve sleep among the employed population, and there is growing evidence that mobile-delivered mindfulness apps such as Calm can serve as a feasible alternative to in-person interventions, with similar benefits. The present results show that not only does a mobile mindfulness app produce benefits for sleep among the working population but that improvements in insomnia in particular may play a key mechanistic role in reducing productivity impairment.

## Appendix

Multimedia Appendix 1 Missing data patterns by week.

Multimedia Appendix 2 Missing data patterns among sleep variables and covariates.

Multimedia Appendix 3 Missing data patterns among productivity variables and covariates.

Multimedia Appendix 4 Mean scores of outcome measures over time by group (all available data).

Multimedia Appendix 5 Growth curve mediation model: insomnia and absenteeism.

## Abbreviations

ISI: Insomnia Severity Index
