# Supplementary material for: The Indirect Effects of a Mindfulness Mobile App on Productivity Through Changes in Sleep Among Retail Employees: Secondary Analysis
Source: JMIR Mhealth Uhealth. 2022 Sep 28;10(9):e40500. doi: 10.2196/40500 (PMC9557984; doi:10.2196/40500)
Supplement: Multimedia Appendix 1 [file mhealth_v10i9e40500_app1.pdf]

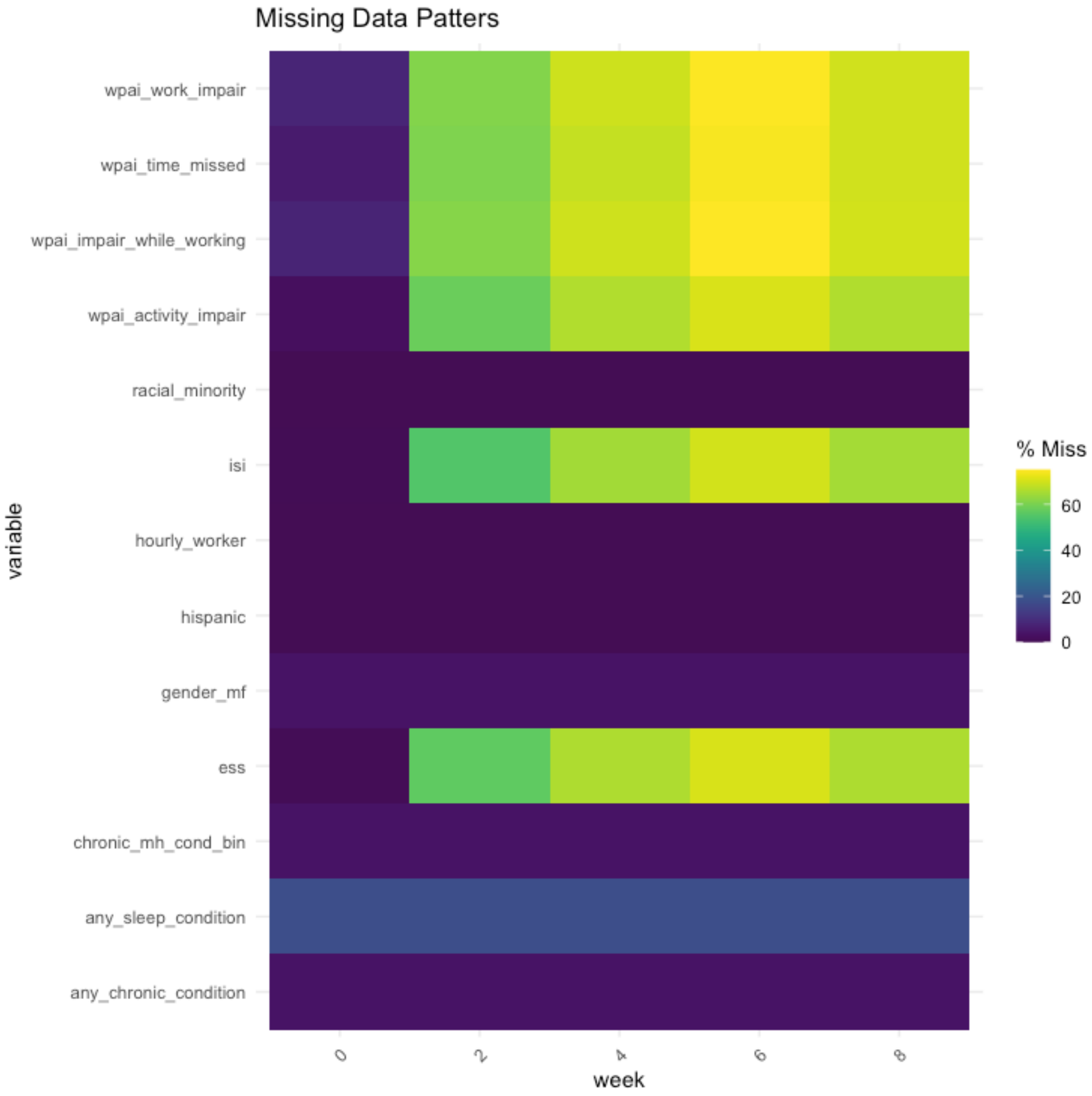

Note. Figure shows missing data patterns for all measured variables included in the growth curve models, at each assessment period
