# Supplementary material for: The Indirect Effects of a Mindfulness Mobile App on Productivity Through Changes in Sleep Among Retail Employees: Secondary Analysis
Source: JMIR Mhealth Uhealth. 2022 Sep 28;10(9):e40500. doi: 10.2196/40500 (PMC9557984; doi:10.2196/40500)
Supplement: Multimedia Appendix 2 [file mhealth_v10i9e40500_app2.pdf]

Missing Data Matrix: Sleep Outcomes

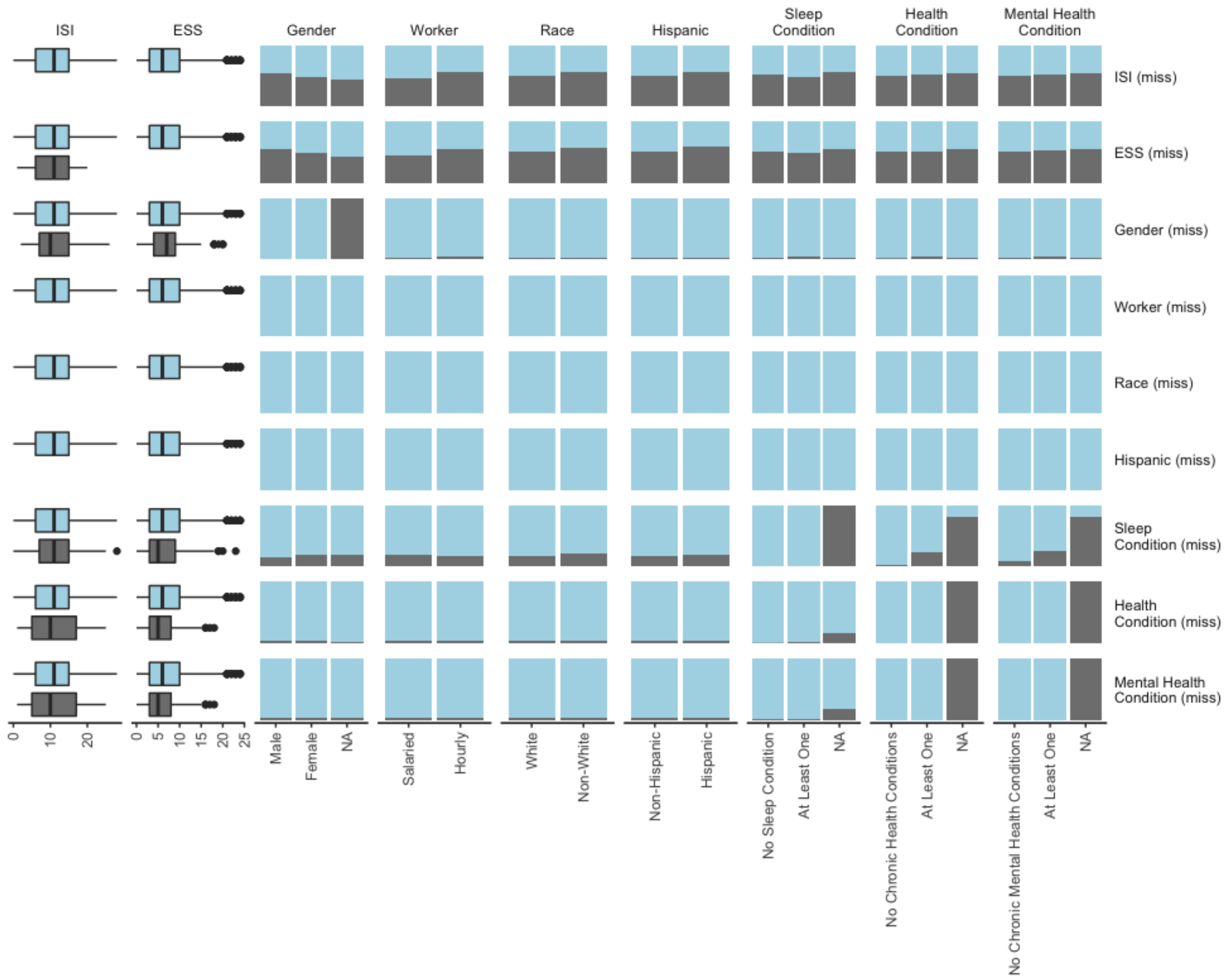

Note. Figure shows missing data patterns in matrix form for covariates and sleep disturbance variables from the growth curve models. Blue shaded elements reflect complete data and gray shaded elements reflect missing data. Bars reflect proportions.
