# Supplementary material for: The Indirect Effects of a Mindfulness Mobile App on Productivity Through Changes in Sleep Among Retail Employees: Secondary Analysis
Source: JMIR Mhealth Uhealth. 2022 Sep 28;10(9):e40500. doi: 10.2196/40500 (PMC9557984; doi:10.2196/40500)
Supplement: Multimedia Appendix 4 [file mhealth_v10i9e40500_app4.pdf]

Outcome	Group	Week 0			Week 2			Week 4			Week 6			Week 8		
		M	*SD*	*N*	*M*	*SD*	*N*	*M*	*SD*	*N*	*M*	*SD*	*N*	*M*	*SD*	*N*
Insomnia symptoms	Waitlist	11.90	5.87	444	11.43	5.68	230	10.93	5.99	182	11.23	6.20	164	11.09	6.58	157
	Calm	11.45	5.65	584	10.51	5.56	235	9.16	5.34	180	8.46	5.92	144	8.56	5.81	203
Daytime sleepiness	Waitlist	7.10	4.61	443	7.33	4.46	220	6.71	4.40	177	6.41	4.61	160	6.62	4.81	154
	Calm	7.26	4.92	582	6.83	4.26	225	6.44	4.76	172	5.55	4.36	141	5.77	4.69	196
Absenteeism (1=yes; 2=no)	Waitlist	0.23	0.42	415	0.14	0.35	203	0.16	0.36	166	0.17	0.38	139	0.18	0.38	136
	Calm	0.19	0.39	559	0.17	0.38	201	0.16	0.37	158	0.17	0.38	124	0.15	0.36	175
Presenteeism	Waitlist	30.62	26.14	406	25.00	25.99	200	27.33	25.12	161	27.01	26.10	134	29.33	27.10	135
	Calm	28.10	25.69	547	25.95	25.07	195	21.51	22.95	152	22.46	25.40	122	21.33	22.54	173
Overall work impairment	Waitlist	33.08	28.44	404	26.87	28.25	202	28.87	26.37	161	30.13	29.20	138	31.53	28.94	136
	Calm	30.30	27.93	546	27.87	26.94	197	23.09	24.80	153	23.41	26.14	120	22.81	24.08	174
Non-work activity impairment	Waitlist	35.87	28.69	438	32.10	29.59	214	30.68	28.46	176	32.83	31.32	159	34.90	30.91	153
	Calm	32.33	27.16	574	31.80	27.74	217	25.92	25.08	169	24.47	26.76	141	24.18	26.12	194
