# Supplementary material for: The Indirect Effects of a Mindfulness Mobile App on Productivity Through Changes in Sleep Among Retail Employees: Secondary Analysis
Source: JMIR Mhealth Uhealth. 2022 Sep 28;10(9):e40500. doi: 10.2196/40500 (PMC9557984; doi:10.2196/40500)
Supplement: Multimedia Appendix 5 [file mhealth_v10i9e40500_app5.pdf]

Latent Variables:

	Estimate	Std.Err	z-value	P(>|z|)	Std.lv	Std.all
y_i ~						
absenteeism, week=0	1				0.247	0.622
absenteeism, week=2	1				0.247	0.647
absenteeism, week=4	1				0.247	0.67
absenteeism, week=6	1				0.247	0.691
absenteeism, week=8	1				0.247	0.71
y_s ~						
absenteeism, week=0	0				0	0
absenteeism, week=2	1				0.029	0.077
absenteeism, week=4	2				0.059	0.159
absenteeism, week=6	3				0.088	0.246
absenteeism, week=8	4				0.117	0.337
m_i ~						
insomnia, week=0	1				5.337	0.921
insomnia, week=2	1				5.337	0.944
insomnia, week=4	1				5.337	0.933
insomnia, week=6	1				5.337	0.892
insomnia, week=8	1				5.337	0.832
m_s ~						

insomnia, week=0	0				0	0
insomnia, week=2	1				1.075	0.19
insomnia, week=4	2				2.151	0.376
insomnia, week=6	3				3.226	0.539
insomnia, week=8	4				4.302	0.671

Regressions:

	Estimate	Std.Err	z-value	P(>|z|)	Std.lv	Std.all
y_i ~						
female	-0.008	0.025	-0.312	0.755	-0.032	-0.016
hourly worker	0.059	0.027	2.16	0.031	0.239	0.117
racial minority	0.031	0.035	0.893	0.372	0.125	0.054
hispanic	0.019	0.042	0.458	0.647	0.078	0.028
chronic sleep dx	0.038	0.04	0.956	0.339	0.154	0.066
any chronic dx	-0.006	0.031	-0.184	0.854	-0.023	-0.012
chronic mh dx	0.218	0.04	5.385	0	0.882	0.411
y_s ~						
female	0.026	0.011	2.391	0.017	0.904	0.452
hourly worker	-0.008	0.012	-0.678	0.498	-0.282	-0.138
racial minority	-0.022	0.014	-1.536	0.125	-0.759	-0.324
hispanic	0.005	0.019	0.25	0.802	0.165	0.06
chronic sleep dx	-0.008	0.016	-0.497	0.619	-0.265	-0.115
any chronic dx	-0.006	0.015	-0.379	0.705	-0.191	-0.096
chronic mh dx	-0.039	0.017	-2.263	0.024	-1.347	-0.628

m_i ~						
female	0.793	0.363	2.182	0.029	0.149	0.074
hourly worker	1.373	0.374	3.671	0	0.257	0.126
racial minority	0.476	0.501	0.951	0.342	0.089	0.038
hispanic	0.742	0.558	1.329	0.184	0.139	0.05
chronic sleep dx	3.375	0.471	7.16	0	0.632	0.274
any chronic dx	0.041	0.515	0.08	0.936	0.008	0.004
chronic mh dx	2.305	0.555	4.156	0	0.432	0.201
m_s ~						
female	-0.236	0.142	-1.663	0.096	-0.219	-0.11
hourly worker	-0.167	0.147	-1.134	0.257	-0.155	-0.076
racial minority	0.301	0.184	1.633	0.102	0.28	0.12
hispanic	0.025	0.228	0.109	0.914	0.023	0.008
chronic sleep dx	-0.278	0.178	-1.562	0.118	-0.259	-0.112
any chronic dx	-0.134	0.185	-0.725	0.468	-0.125	-0.062
chronic mh dx	-0.028	0.198	-0.139	0.889	-0.026	-0.012
y_s ~						
group (c)	0.007	0.01	0.706	0.48	0.243	0.12
m_s ~						
group (a)	-0.56	0.128	-4.372	0	-0.521	-0.257
y_s ~						
m_s (b)	0.009	0.006	1.406	0.16	0.317	0.317

Covariances:

	Estimate	Std.Err	z-value	P(>|z|)	Std.lv	Std.all
y_i ~~						
y_s	-0.004	0.003	-1.301	0.193	-1.262	-1.262
m_i ~~						
m_s	-1.2	0.365	-3.288	0.001	-0.246	-0.246
y_i ~~						
m_i	0				0	0
m_s	0				0	0
y_s ~~						
m_i	0				0	0
m_s	0				0	0

Intercepts:

	Estimate	Std.Err	z-value	P(>|z|)	Std.lv	Std.all
absenteeism, week=0	0				0	0
absenteeism, week=2	0				0	0
absenteeism, week=4	0				0	0
absenteeism, week=6	0				0	0
absenteeism, week=8	0				0	0
insomnia, week=0	0				0	0
insomnia, week=2	0				0	0
insomnia, week=4	0				0	0
insomnia, week=6	0				0	0
insomnia, week=8	0				0	0

y_i	0.078	0.061	1.269	0.205	0.314	0.314
y_s	0.009	0.029	0.306	0.76	0.301	0.301
m_i	8.476	0.962	8.808	0	1.588	1.588
m_s	0.261	0.375	0.698	0.485	0.243	0.243

Variances:

	Estimate	Std.Err	z-value	P(>|z|)	Std.lv	Std.all
absenteeism, week=0	0.096	0.007	13.072	0	0.096	0.613
absenteeism, week=2	0.096	0.007	13.072	0	0.096	0.662
absenteeism, week=4	0.096	0.007	13.072	0	0.096	0.71
absenteeism, week=6	0.096	0.007	13.072	0	0.096	0.756
absenteeism, week=8	0.096	0.007	13.072	0	0.096	0.797
insomnia, week=0	5.092	0.287	17.754	0	5.092	0.152
insomnia, week=2	5.092	0.287	17.754	0	5.092	0.159
insomnia, week=4	5.092	0.287	17.754	0	5.092	0.156
insomnia, week=6	5.092	0.287	17.754	0	5.092	0.142
insomnia, week=8	5.092	0.287	17.754	0	5.092	0.124
y_i	0.047	0.009	5.236	0	0.763	0.763
y_s	0	0.001	0.144	0.886	0.234	0.234
m_i	22.936	1.229	18.669	0	0.805	0.805
m_s	1.041	0.159	6.54	0	0.9	0.9

Growth Curve Mediation Model: Daytime Sleepiness and Absenteeism

Latent Variables:

	Estimate	Std.Err	z-value	P(>|z|)	Std.lv	Std.all

y_i =~			
absenteeism, week=0	1	0.247	0.622
absenteeism, week=2	1	0.247	0.647
absenteeism, week=4	1	0.247	0.67
absenteeism, week=6	1	0.247	0.691
absenteeism, week=8	1	0.247	0.709
y_s =~			
absenteeism, week=0	0	0	0
absenteeism, week=2	1	0.03	0.079
absenteeism, week=4	2	0.06	0.164
absenteeism, week=6	3	0.09	0.253
absenteeism, week=8	4	0.121	0.346
m_i =~			
sleepiness, week=0	1	4.418	0.927
sleepiness, week=2	1	4.418	0.946
sleepiness, week=4	1	4.418	0.947
sleepiness, week=6	1	4.418	0.93
sleepiness, week=8	1	4.418	0.898
m_s =~			
sleepiness, week=0	0	0	0
sleepiness, week=2	1	0.653	0.14
sleepiness, week=4	2	1.306	0.28
sleepiness, week=6	3	1.959	0.412
sleepiness, week=8	4	2.612	0.531

Regressions:

	Estimate	Std.Err	z-value	P(>|z|)	Std.lv	Std.all
y_i ~						
female	-0.008	0.025	-0.323	0.747	-0.033	-0.016
hourly worker	0.059	0.026	2.282	0.023	0.239	0.117
racial minority	0.031	0.035	0.881	0.378	0.126	0.054
hispanic	0.02	0.043	0.458	0.647	0.079	0.029
chronic sleep dx	0.038	0.04	0.943	0.345	0.153	0.066
any chronic dx	-0.006	0.031	-0.2	0.842	-0.025	-0.013
chronic mh dx	0.217	0.039	5.619	0	0.88	0.41
y_s ~						
female	0.024	0.011	2.212	0.027	0.81	0.405
hourly worker	-0.009	0.012	-0.736	0.462	-0.284	-0.138
racial minority	-0.022	0.014	-1.528	0.127	-0.716	-0.306
hispanic	0.007	0.02	0.363	0.716	0.24	0.087
chronic sleep dx	-0.009	0.015	-0.587	0.557	-0.297	-0.128
any chronic dx	-0.006	0.015	-0.378	0.706	-0.186	-0.093
chronic mh dx	-0.039	0.017	-2.218	0.027	-1.278	-0.596
m_i ~						
female	0.221	0.33	0.67	0.503	0.05	0.025
hourly worker	0.786	0.338	2.329	0.02	0.178	0.087
racial minority	0.144	0.398	0.363	0.717	0.033	0.014

hispanic	0.708	0.468	1.512	0.13	0.16	0.058
chronic sleep dx	1.317	0.436	3.021	0.003	0.298	0.129
any chronic dx	-0.376	0.475	-0.792	0.429	-0.085	-0.043
chronic mh dx	0.443	0.517	0.857	0.392	0.1	0.047
m_s ~						
female	-0.067	0.101	-0.663	0.507	-0.103	-0.051
hourly worker	0.05	0.098	0.506	0.613	0.076	0.037
racial minority	-0.051	0.12	-0.428	0.669	-0.079	-0.034
hispanic	0.248	0.176	1.415	0.157	0.38	0.138
chronic sleep dx	0.112	0.122	0.919	0.358	0.172	0.074
any chronic dx	0.086	0.13	0.658	0.511	0.131	0.066
chronic mh dx	0.102	0.142	0.716	0.474	0.156	0.073
y_s ~						
group (c)	-0.001	0.01	-0.089	0.929	-0.03	-0.015
m_s ~						
group (a)	-0.261	0.092	-2.841	0.005	-0.4	-0.197
y_s ~						
m_s (b)	-0.011	0.019	-0.578	0.563	-0.24	-0.24

Covariances:

	Estimate	Std.Err	z-value	P(>|z|)	Std.lv	Std.all
y_i ~~						
y_s	-0.004	0.003	-1.321	0.187	-1.115	-1.115
m_i ~~						

m_s	-0.749	0.271	-2.762	0.006	-0.277	-0.277
y_i ~~						
m_i	0				0	0
m_s	0				0	0
y_s ~~						
m_i	0				0	0
m_s	0				0	0

Intercepts:

	Estimate	Std.Err	z-value	P(>|z|)	Std.lv	Std.all
absenteeism, week=0	0				0	0
absenteeism, week=2	0				0	0
absenteeism, week=4	0				0	0
absenteeism, week=6	0				0	0
absenteeism, week=8	0				0	0
sleepiness, week=0	0				0	0
sleepiness, week=2	0				0	0
sleepiness, week=4	0				0	0
sleepiness, week=6	0				0	0
sleepiness, week=8	0				0	0
y_i	0.078	0.061	1.293	0.196	0.317	0.317
y_s	0.006	0.03	0.215	0.83	0.211	0.211
m_i	6.677	0.887	7.532	0	1.511	1.511
m_s	-0.334	0.252	-1.322	0.186	-0.511	-0.511

Variances:

	Estimate	Std.Err	z-value	P(>|z|)	Std.lv	Std.all
absenteeism, week=0	0.096	0.008	12.629	0	0.096	0.613
absenteeism, week=2	0.096	0.008	12.629	0	0.096	0.662
absenteeism, week=4	0.096	0.008	12.629	0	0.096	0.711
absenteeism, week=6	0.096	0.008	12.629	0	0.096	0.756
absenteeism, week=8	0.096	0.008	12.629	0	0.096	0.795
sleepiness, week=0	3.216	0.29	11.079	0	3.216	0.141
sleepiness, week=2	3.216	0.29	11.079	0	3.216	0.147
sleepiness, week=4	3.216	0.29	11.079	0	3.216	0.148
sleepiness, week=6	3.216	0.29	11.079	0	3.216	0.143
sleepiness, week=8	3.216	0.29	11.079	0	3.216	0.133
y_i	0.047	0.009	5.13	0	0.763	0.763
y_s	0	0.001	0.188	0.851	0.294	0.294
m_i	18.581	1.111	16.732	0	0.952	0.952
m_s	0.394	0.086	4.581	0	0.925	0.925

Growth Curve Mediation Model: Insomnia and Presenteeism

Latent Variables:

	Estimate	Std.Err	z-value	P(>|z|)	Std.lv	Std.all
y_i =~						
presenteeism, week=0	1				20.519	0.796
presenteeism, week=2	1				20.519	0.82

presenteeism, week=4	1	20.519	0.822
presenteeism, week=6	1	20.519	0.801
presenteeism, week=8	1	20.519	0.763
y_s =~			
presenteeism, week=0	0	0	0
presenteeism, week=2	1	4.169	0.167
presenteeism, week=4	2	8.339	0.334
presenteeism, week=6	3	12.508	0.488
presenteeism, week=8	4	16.677	0.62
m_i =~			
insomnia, week=0	1	5.246	0.917
insomnia, week=2	1	5.246	0.926
insomnia, week=4	1	5.246	0.904
insomnia, week=6	1	5.246	0.857
insomnia, week=8	1	5.246	0.795
m_s =~			
insomnia, week=0	0	0	0
insomnia, week=2	1	1.058	0.187
insomnia, week=4	2	2.117	0.365
insomnia, week=6	3	3.175	0.519
insomnia, week=8	4	4.233	0.642

Regressions:

	Estimate	Std.Err	z-value	P(>|z|)	Std.lv	Std.all
y_i ~						
female	-0.088	1.643	-0.054	0.957	-0.004	-0.002
hourly worker	7.326	1.67	4.387	0	0.357	0.174
racial minority	4.217	2.102	2.006	0.045	0.206	0.088
hispanic	2.218	2.687	0.825	0.409	0.108	0.039
chronic sleep dx	5.156	2.189	2.355	0.019	0.251	0.109
any chronic dx	-3.748	2.208	-1.698	0.09	-0.183	-0.091
chronic mh dx	16.646	2.516	6.616	0	0.811	0.378
y_s ~						
female	2.055	0.714	2.877	0.004	0.493	0.246
hourly worker	-0.503	0.741	-0.68	0.497	-0.121	-0.059
racial minority	-1.132	0.876	-1.293	0.196	-0.272	-0.116
hispanic	0.862	1.092	0.79	0.43	0.207	0.075
chronic sleep dx	1.632	0.916	1.782	0.075	0.391	0.169
any chronic dx	0.404	0.994	0.407	0.684	0.097	0.048
chronic mh dx	-1.274	0.965	-1.32	0.187	-0.306	-0.142
m_i ~						
female	0.794	0.373	2.131	0.033	0.151	0.076
hourly worker	1.375	0.361	3.804	0	0.262	0.128
racial minority	0.478	0.493	0.969	0.333	0.091	0.039
hispanic	0.741	0.555	1.337	0.181	0.141	0.051
chronic sleep dx	3.372	0.489	6.899	0	0.643	0.278
any chronic dx	0.034	0.544	0.063	0.95	0.007	0.003

chronic mh dx		2.303	0.556	4.139	0	0.439	0.205
m_s ~							
female		-0.241	0.138	-1.745	0.081	-0.228	-0.114
hourly worker		-0.165	0.141	-1.17	0.242	-0.156	-0.076
racial minority		0.301	0.176	1.715	0.086	0.285	0.122
hispanic		0.026	0.223	0.115	0.908	0.024	0.009
chronic sleep dx		-0.283	0.176	-1.611	0.107	-0.267	-0.116
any chronic dx		-0.12	0.19	-0.633	0.527	-0.114	-0.057
chronic mh dx		-0.02	0.196	-0.1	0.92	-0.019	-0.009
y_s ~							
group	(c)	0.848	0.74	1.145	0.252	0.203	0.1
m_s ~							
group	(a)	-0.553	0.125	-4.439	0	-0.523	-0.258
y_s ~							
m_s	(b)	3.236	0.798	4.056	0	0.821	0.821

Covariances:

	Estimate	Std.Err	z-value	P(>|z|)	Std.lv	Std.all
y_i ~~						
y_s	-21.084	10.238	-2.059	0.039	-0.526	-0.526
m_i ~~						
m_s	-0.71	0.464	-1.529	0.126	-0.151	-0.151
y_i ~~						
m_i	0				0	0

m_s	0				0	0
y_s ~~						
m_i	0				0	0
m_s	0				0	0

Intercepts:

	Estimate	Std.Err	z-value	P(>|z|)	Std.lv	Std.all
presenteeism, week=0	0				0	0
presenteeism, week=2	0				0	0
presenteeism, week=4	0				0	0
presenteeism, week=6	0				0	0
presenteeism, week=8	0				0	0
insomnia, week=0	0				0	0
insomnia, week=2	0				0	0
insomnia, week=4	0				0	0
insomnia, week=6	0				0	0
insomnia, week=8	0				0	0
y_i	21.293	4.125	5.162	0	1.038	1.038
y_s	-1.382	1.921	-0.72	0.472	-0.331	-0.331
m_i	8.491	1.012	8.392	0	1.619	1.619
m_s	0.227	0.382	0.593	0.553	0.214	0.214

Variances:

	Estimate	Std.Err	z-value	P(>|z|)	Std.lv	Std.all

presenteeism, week=0	242.959	24.245	10.021	0	242.959	0.366
presenteeism, week=2	242.959	24.245	10.021	0	242.959	0.388
presenteeism, week=4	242.959	24.245	10.021	0	242.959	0.389
presenteeism, week=6	242.959	24.245	10.021	0	242.959	0.37
presenteeism, week=8	242.959	24.245	10.021	0	242.959	0.336
insomnia, week=0	5.2	0.346	15.049	0	5.2	0.159
insomnia, week=2	5.2	0.346	15.049	0	5.2	0.162
insomnia, week=4	5.2	0.346	15.049	0	5.2	0.155
insomnia, week=6	5.2	0.346	15.049	0	5.2	0.139
insomnia, week=8	5.2	0.346	15.049	0	5.2	0.119
y_i	285.612	29.866	9.563	0	0.678	0.678
y_s	5.624	4.402	1.278	0.201	0.324	0.324
m_i	21.965	1.324	16.588	0	0.798	0.798
m_s	1.005	0.173	5.796	0	0.897	0.897

Growth Curve Mediation Model: Daytime Sleepiness and Presenteeism

Latent Variables:

	Estimate	Std.Err	z-value	P(>|z|)	Std.lv	Std.all
y_i =~						
presenteeism, week=0	1				20.484	0.795
presenteeism, week=2	1				20.484	0.819
presenteeism, week=4	1				20.484	0.821
presenteeism, week=6	1				20.484	0.801
presenteeism, week=8	1				20.484	0.764
y_s =~						

presenteeism, week=0	0				0	0
presenteeism, week=2	1				4.122	0.165
presenteeism, week=4	2				8.244	0.33
presenteeism, week=6	3				12.365	0.484
presenteeism, week=8	4				16.487	0.615
m_i =~						
sleepiness, week=0	1				4.398	0.926
sleepiness, week=2	1				4.398	0.941
sleepiness, week=4	1				4.398	0.938
sleepiness, week=6	1				4.398	0.918
sleepiness, week=8	1				4.398	0.883
m_s =~						
sleepiness, week=0	0				0	0
sleepiness, week=2	1				0.655	0.14
sleepiness, week=4	2				1.309	0.279
sleepiness, week=6	3				1.964	0.41
sleepiness, week=8	4				2.618	0.526

Regressions:

	Estimate	Std.Err	z-value	P(>|z|)	Std.lv	Std.all
y_i ~						
female	-0.126	1.611	-0.078	0.938	-0.006	-0.003
hourly worker	7.286	1.63	4.47	0	0.356	0.174

racial minority	4.258	2.142	1.988	0.047	0.208	0.089
hispanic	2.245	2.662	0.843	0.399	0.11	0.04
chronic sleep dx	5.155	2.213	2.329	0.02	0.252	0.109
any chronic dx	-3.852	2.28	-1.69	0.091	-0.188	-0.094
chronic mh dx	16.597	2.438	6.809	0	0.81	0.378
y_s ~						
female	1.29	0.901	1.432	0.152	0.313	0.156
hourly worker	-0.859	1.058	-0.812	0.417	-0.208	-0.102
racial minority	-0.17	1.246	-0.137	0.891	-0.041	-0.018
hispanic	0.669	1.479	0.452	0.651	0.162	0.059
chronic sleep dx	0.428	1.192	0.359	0.719	0.104	0.045
any chronic dx	0.017	1.197	0.015	0.988	0.004	0.002
chronic mh dx	-1.377	1.22	-1.129	0.259	-0.334	-0.156
m_i ~						
female	0.221	0.328	0.674	0.5	0.05	0.025
hourly worker	0.786	0.338	2.327	0.02	0.179	0.087
racial minority	0.145	0.409	0.353	0.724	0.033	0.014
hispanic	0.708	0.475	1.489	0.137	0.161	0.058
chronic sleep dx	1.316	0.453	2.904	0.004	0.299	0.129
any chronic dx	-0.377	0.487	-0.774	0.439	-0.086	-0.043
chronic mh dx	0.443	0.515	0.86	0.39	0.101	0.047
m_s ~						
female	-0.066	0.102	-0.651	0.515	-0.101	-0.051
hourly worker	0.049	0.101	0.483	0.629	0.075	0.036

racial minority	-0.05	0.119	-0.42	0.674	-0.076	-0.033
hispanic	0.249	0.171	1.457	0.145	0.381	0.138
chronic sleep dx	0.115	0.122	0.942	0.346	0.175	0.076
any chronic dx	0.087	0.133	0.655	0.512	0.133	0.066
chronic mh dx	0.099	0.144	0.687	0.492	0.151	0.07
y_s ~						
group (c)	-0.615	1.837	-0.335	0.738	-0.149	-0.074
m_s ~						
group (a)	-0.257	0.094	-2.725	0.006	-0.393	-0.194
y_s ~						
m_s (b)	1.103	6.14	0.18	0.857	0.175	0.175

Covariances:

	Estimate	Std.Err	z-value	P(>|z|)	Std.lv	Std.all
y_i ~~						
y_s	-20.774	10.658	-1.949	0.051	-0.314	-0.314
m_i ~~						
m_s	-0.654	0.313	-2.092	0.036	-0.242	-0.242
y_i ~~						
m_i	0				0	0
m_s	0				0	0
y_s ~~						
m_i	0				0	0
m_s	0				0	0

Intercepts:

	Estimate	Std.Err	z-value	P(>|z|)	Std.lv	Std.all
presenteeism, week=0	0				0	0
presenteeism, week=2	0				0	0
presenteeism, week=4	0				0	0
presenteeism, week=6	0				0	0
presenteeism, week=8	0				0	0
sleepiness, week=0	0				0	0
sleepiness, week=2	0				0	0
sleepiness, week=4	0				0	0
sleepiness, week=6	0				0	0
sleepiness, week=8	0				0	0
y_i	21.476	4.214	5.097	0	1.048	1.048
y_s	-0.552	2.218	-0.249	0.803	-0.134	-0.134
m_i	6.681	0.91	7.34	0	1.519	1.519
m_s	-0.34	0.262	-1.298	0.194	-0.52	-0.52

Variances:

	Estimate	Std.Err	z-value	P(>|z|)	Std.lv	Std.all
presenteeism, week=0	243.893	23.174	10.524	0	243.893	0.368
presenteeism, week=2	243.893	23.174	10.524	0	243.893	0.389
presenteeism, week=4	243.893	23.174	10.524	0	243.893	0.391
presenteeism, week=6	243.893	23.174	10.524	0	243.893	0.373

presenteeism, week=8	243.893	23.174	10.524	0	243.893	0.339
sleepiness, week=0	3.214	0.294	10.942	0	3.214	0.143
sleepiness, week=2	3.214	0.294	10.942	0	3.214	0.147
sleepiness, week=4	3.214	0.294	10.942	0	3.214	0.146
sleepiness, week=6	3.214	0.294	10.942	0	3.214	0.14
sleepiness, week=8	3.214	0.294	10.942	0	3.214	0.13
y_i	283.945	30.204	9.401	0	0.677	0.677
y_s	15.423	6.393	2.413	0.016	0.908	0.908
m_i	18.399	1.148	16.022	0	0.951	0.951
m_s	0.397	0.087	4.544	0	0.927	0.927

Growth Curve Mediation Model: Insomnia and Work Impairment

Latent Variables:

	Estimate	Std.Err	z-value	P(>|z|)	Std.lv	Std.all
y_i =~						
impairment, week=0	1				21.947	0.788
impairment, week=2	1				21.947	0.805
impairment, week=4	1				21.947	0.806
impairment, week=6	1				21.947	0.789
impairment, week=8	1				21.947	0.759
y_s =~						
impairment, week=0	0				0	0
impairment, week=2	1				4.021	0.148
impairment, week=4	2				8.042	0.295
impairment, week=6	3				12.064	0.434

impairment, week=8	4				16.085	0.556
m_i =~						
insomnia, week=0	1				5.254	0.918
insomnia, week=2	1				5.254	0.928
insomnia, week=4	1				5.254	0.907
insomnia, week=6	1				5.254	0.86
insomnia, week=8	1				5.254	0.798
m_s =~						
insomnia, week=0	0				0	0
insomnia, week=2	1				1.06	0.187
insomnia, week=4	2				2.121	0.366
insomnia, week=6	3				3.181	0.521
insomnia, week=8	4				4.241	0.644

Regressions:

	Estimate	Std.Err	z-value	P(>|z|)	Std.lv	Std.all
y_i ~						
female	0.292	1.756	0.166	0.868	0.013	0.007
hourly worker	9.008	1.718	5.244	0	0.41	0.2
racial minority	4.859	2.413	2.013	0.044	0.221	0.095
hispanic	2.446	2.817	0.868	0.385	0.111	0.04
chronic sleep dx	5.572	2.422	2.3	0.021	0.254	0.11
any chronic dx	-3.694	2.34	-1.579	0.114	-0.168	-0.084

chronic mh dx	18.486	2.567	7.201	0	0.842	0.393
y_s ~						
female	2.067	0.768	2.691	0.007	0.514	0.257
hourly worker	-0.63	0.749	-0.841	0.4	-0.157	-0.076
racial minority	-1.333	0.909	-1.466	0.143	-0.331	-0.142
hispanic	0.593	1.151	0.515	0.607	0.147	0.053
chronic sleep dx	1.508	1.041	1.449	0.147	0.375	0.162
any chronic dx	0.331	1.052	0.315	0.753	0.082	0.041
chronic mh dx	-1.339	1.018	-1.314	0.189	-0.333	-0.155
m_i ~						
female	0.792	0.381	2.079	0.038	0.151	0.075
hourly worker	1.374	0.363	3.789	0	0.261	0.128
racial minority	0.479	0.495	0.968	0.333	0.091	0.039
hispanic	0.741	0.557	1.331	0.183	0.141	0.051
chronic sleep dx	3.372	0.494	6.825	0	0.642	0.278
any chronic dx	0.035	0.486	0.073	0.942	0.007	0.003
chronic mh dx	2.307	0.523	4.413	0	0.439	0.205
m_s ~						
female	-0.234	0.141	-1.656	0.098	-0.221	-0.11
hourly worker	-0.161	0.139	-1.156	0.248	-0.152	-0.074
racial minority	0.298	0.186	1.599	0.11	0.281	0.12
hispanic	0.025	0.222	0.114	0.909	0.024	0.009
chronic sleep dx	-0.278	0.166	-1.672	0.095	-0.262	-0.114
any chronic dx	-0.123	0.184	-0.671	0.502	-0.116	-0.058

chronic mh dx	-0.036	0.198	-0.182	0.856	-0.034	-0.016
y_s ~						
group (c)	0.708	0.855	0.829	0.407	0.176	0.087
m_s ~						
group (a)	-0.552	0.127	-4.353	0	-0.52	-0.257
y_s ~						
m_s (b)	3.129	0.893	3.506	0	0.825	0.825

Covariances:

	Estimate	Std.Err	z-value	P(>|z|)	Std.lv	Std.all
y_i ~~						
y_s	-16.47	11.702	-1.408	0.159	-0.42	-0.42
m_i ~~						
m_s	-0.751	0.465	-1.615	0.106	-0.159	-0.159
y_i ~~						
m_i	0				0	0
m_s	0				0	0
y_s ~~						
m_i	0				0	0
m_s	0				0	0

Intercepts:

	Estimate	Std.Err	z-value	P(>|z|)	Std.lv	Std.all
impairment, week=0	0				0	0

impairment, week=2	0				0	0
impairment, week=4	0				0	0
impairment, week=6	0				0	0
impairment, week=8	0				0	0
insomnia, week=0	0				0	0
insomnia, week=2	0				0	0
insomnia, week=4	0				0	0
insomnia, week=6	0				0	0
insomnia, week=8	0				0	0
y_i	21.003	4.452	4.718	0	0.957	0.957
y_s	-1.046	2.026	-0.517	0.605	-0.26	-0.26
m_i	8.489	0.93	9.125	0	1.616	1.616
m_s	0.23	0.37	0.622	0.534	0.217	0.217

Variances:

	Estimate	Std.Err	z-value	P(>|z|)	Std.lv	Std.all
impairment, week=0	294.113	26.108	11.265	0	294.113	0.379
impairment, week=2	294.113	26.108	11.265	0	294.113	0.396
impairment, week=4	294.113	26.108	11.265	0	294.113	0.397
impairment, week=6	294.113	26.108	11.265	0	294.113	0.381
impairment, week=8	294.113	26.108	11.265	0	294.113	0.352
insomnia, week=0	5.186	0.33	15.719	0	5.186	0.158
insomnia, week=2	5.186	0.33	15.719	0	5.186	0.162
insomnia, week=4	5.186	0.33	15.719	0	5.186	0.155

insomnia, week=6	5.186	0.33	15.719	0	5.186	0.139
insomnia, week=8	5.186	0.33	15.719	0	5.186	0.12
y_i	313.683	32.756	9.576	0	0.651	0.651
y_s	4.905	5.05	0.971	0.331	0.303	0.303
m_i	22.048	1.34	16.454	0	0.799	0.799
m_s	1.011	0.173	5.838	0	0.899	0.899

Growth Curve Mediation Model: Daytime Sleepiness and Work Impairment

Latent Variables:

	Estimate	Std.Err	z-value	P(>|z|)	Std.lv	Std.all
y_i =~						
impairment, week=0	1				21.904	0.787
Impairment.2	1				21.904	0.804
Impairment.4	1				21.904	0.805
impairment, week=6	1				21.904	0.789
impairment, week=8	1				21.904	0.76
y_s =~						
impairment, week=0	0				0	0
Impairment.2	1				3.964	0.146
Impairment.4	2				7.928	0.291
impairment, week=6	3				11.892	0.429
impairment, week=8	4				15.856	0.55
m_i =~						
sleepiness, week=0	1				4.398	0.926
sleepiness, week=2	1				4.398	0.941

sleepiness, week=4	1				4.398	0.939
sleepiness, week=6	1				4.398	0.918
sleepiness, week=8	1				4.398	0.883
m_s =~						
sleepiness, week=0	0				0	0
sleepiness, week=2	1				0.655	0.14
sleepiness, week=4	2				1.309	0.279
sleepiness, week=6	3				1.964	0.41
sleepiness, week=8	4				2.619	0.526

Regressions:

	Estimate	Std.Err	z-value	P(>|z|)	Std.lv	Std.all
y_i ~						
female	0.229	1.718	0.133	0.894	0.01	0.005
hourly worker	8.973	1.814	4.946	0	0.41	0.2
racial minority	4.871	2.27	2.146	0.032	0.222	0.095
hispanic	2.469	2.942	0.839	0.401	0.113	0.041
chronic sleep dx	5.535	2.325	2.38	0.017	0.253	0.109
any chronic dx	-3.799	2.494	-1.523	0.128	-0.173	-0.087
chronic mh dx	18.455	2.618	7.049	0	0.843	0.393
y_s ~						
female	1.383	0.837	1.653	0.098	0.349	0.174
hourly worker	-0.954	1.117	-0.854	0.393	-0.241	-0.117

racial minority	-0.39	1.484	-0.263	0.793	-0.098	-0.042
hispanic	0.439	2.015	0.218	0.827	0.111	0.04
chronic sleep dx	0.401	1.242	0.323	0.747	0.101	0.044
any chronic dx	-0.031	1.102	-0.028	0.978	-0.008	-0.004
chronic mh dx	-1.503	1.38	-1.089	0.276	-0.379	-0.177
m_i ~						
female	0.221	0.334	0.661	0.508	0.05	0.025
hourly worker	0.786	0.326	2.413	0.016	0.179	0.087
racial minority	0.145	0.411	0.352	0.725	0.033	0.014
hispanic	0.708	0.476	1.488	0.137	0.161	0.058
chronic sleep dx	1.316	0.456	2.883	0.004	0.299	0.129
any chronic dx	-0.377	0.48	-0.784	0.433	-0.086	-0.043
chronic mh dx	0.444	0.514	0.862	0.388	0.101	0.047
m_s ~						
female	-0.066	0.1	-0.653	0.513	-0.1	-0.05
hourly worker	0.05	0.099	0.499	0.618	0.076	0.037
racial minority	-0.051	0.122	-0.416	0.677	-0.077	-0.033
hispanic	0.249	0.183	1.366	0.172	0.381	0.138
chronic sleep dx	0.115	0.122	0.945	0.344	0.176	0.076
any chronic dx	0.087	0.128	0.676	0.499	0.132	0.066
chronic mh dx	0.097	0.143	0.678	0.498	0.148	0.069
y_s ~						
group (c)	-0.72	2.83	-0.254	0.799	-0.182	-0.09
m_s ~						

group	(a)	-0.257	0.098	-2.614	0.009	-0.393	-0.194
y_s ~							
m_s	(b)	0.978	6.853	0.143	0.887	0.162	0.162

Covariances:

	Estimate	Std.Err	z-value	P(>|z|)	Std.lv	Std.all
y_i ~~						
y_s	-16.241	11.707	-1.387	0.165	-0.245	-0.245
m_i ~~						
m_s	-0.658	0.304	-2.167	0.03	-0.243	-0.243
y_i ~~						
m_i	0				0	0
m_s	0				0	0
y_s ~~						
m_i	0				0	0
m_s	0				0	0

Intercepts:

	Estimate	Std.Err	z-value	P(>|z|)	Std.lv	Std.all
impairment, week=0	0				0	0
impairment, week=2	0				0	0
impairment, week=4	0				0	0
impairment, week=6	0				0	0
impairment, week=8	0				0	0

sleepiness, week=0	0				0	0
sleepiness, week=2	0				0	0
sleepiness, week=4	0				0	0
sleepiness, week=6	0				0	0
sleepiness, week=8	0				0	0
y_i	21.211	4.589	4.622	0	0.968	0.968
y_s	-0.321	2.427	-0.132	0.895	-0.081	-0.081
m_i	6.68	0.911	7.334	0	1.519	1.519
m_s	-0.34	0.255	-1.331	0.183	-0.519	-0.519

Variances:

	Estimate	Std.Err	z-value	P(>|z|)	Std.lv	Std.all
impairment, week=0	295.423	26.752	11.043	0	295.423	0.381
impairment, week=2	295.423	26.752	11.043	0	295.423	0.398
impairment, week=4	295.423	26.752	11.043	0	295.423	0.399
impairment, week=6	295.423	26.752	11.043	0	295.423	0.384
impairment, week=8	295.423	26.752	11.043	0	295.423	0.356
sleepiness, week=0	3.214	0.299	10.757	0	3.214	0.142
sleepiness, week=2	3.214	0.299	10.757	0	3.214	0.147
sleepiness, week=4	3.214	0.299	10.757	0	3.214	0.146
sleepiness, week=6	3.214	0.299	10.757	0	3.214	0.14
sleepiness, week=8	3.214	0.299	10.757	0	3.214	0.13
y_i	311.597	34.013	9.161	0	0.649	0.649
y_s	14.09	6.136	2.296	0.022	0.897	0.897

m_i	18.407	1.118	16.46	0	0.951	0.951
m_s	0.397	0.086	4.635	0	0.927	0.927

Growth Curve Mediation Model: Insomnia and Activity Impairment

Latent Variables:

	Estimate	Std.Err	z-value	P(>|z|)	Std.lv	Std.all
y_i =~						
impairment, week=0	1				23.212	0.826
impairment, week=2	1				23.212	0.844
impairment, week=4	1				23.212	0.839
impairment, week=6	1				23.212	0.812
impairment, week=8	1				23.212	0.77
y_s =~						
impairment, week=0	0				0	0
impairment, week=2	1				4.577	0.166
impairment, week=4	2				9.155	0.331
impairment, week=6	3				13.732	0.481
impairment, week=8	4				18.31	0.607
m_i =~						
insomnia, week=0	1				5.261	0.918
insomnia, week=2	1				5.261	0.929
insomnia, week=4	1				5.261	0.909
insomnia, week=6	1				5.261	0.863
insomnia, week=8	1				5.261	0.801
m_s =~						

insomnia, week=0	0				0	0
insomnia, week=2	1				1.061	0.187
insomnia, week=4	2				2.123	0.367
insomnia, week=6	3				3.184	0.522
insomnia, week=8	4				4.245	0.646

Regressions:

	Estimate	Std.Err	z-value	P(>|z|)	Std.lv	Std.all
y_i ~						
female	4.576	1.739	2.632	0.008	0.197	0.099
hourly worker	9.394	1.807	5.197	0	0.405	0.197
racial minority	3.129	2.268	1.379	0.168	0.135	0.058
hispanic	0.602	2.789	0.216	0.829	0.026	0.009
chronic sleep dx	7.672	2.508	3.06	0.002	0.331	0.143
any chronic dx	-4.013	2.469	-1.625	0.104	-0.173	-0.086
chronic mh dx	17.865	2.687	6.65	0	0.77	0.359
y_s ~						
female	0.394	0.746	0.528	0.598	0.086	0.043
hourly worker	-0.865	0.738	-1.173	0.241	-0.189	-0.092
racial minority	0.199	1.038	0.192	0.848	0.044	0.019
hispanic	0.509	0.956	0.533	0.594	0.111	0.04
chronic sleep dx	1.146	1.042	1.1	0.271	0.25	0.108
any chronic dx	-0.066	0.964	-0.068	0.945	-0.014	-0.007

chronic mh dx	-0.623	0.973	-0.64	0.522	-0.136	-0.063
m_i ~						
female	0.797	0.365	2.185	0.029	0.151	0.076
hourly worker	1.379	0.382	3.61	0	0.262	0.128
racial minority	0.483	0.49	0.986	0.324	0.092	0.039
hispanic	0.732	0.573	1.279	0.201	0.139	0.05
chronic sleep dx	3.376	0.474	7.122	0	0.642	0.278
any chronic dx	0.04	0.546	0.073	0.942	0.008	0.004
chronic mh dx	2.312	0.544	4.252	0	0.44	0.205
m_s ~						
female	-0.244	0.144	-1.692	0.091	-0.23	-0.115
hourly worker	-0.17	0.14	-1.213	0.225	-0.16	-0.078
racial minority	0.3	0.184	1.628	0.103	0.282	0.121
hispanic	0.045	0.222	0.205	0.838	0.043	0.016
chronic sleep dx	-0.29	0.174	-1.674	0.094	-0.274	-0.118
any chronic dx	-0.142	0.18	-0.786	0.432	-0.133	-0.067
chronic mh dx	-0.048	0.201	-0.236	0.813	-0.045	-0.021
y_s ~						
group (c)	0.109	0.754	0.145	0.885	0.024	0.012
m_s ~						
group (a)	-0.553	0.129	-4.298	0	-0.521	-0.257
y_s ~						
m_s (b)	3.173	0.705	4.503	0	0.736	0.736

Covariances:

	Estimate	Std.Err	z-value	P(>|z|)	Std.lv	Std.all
y_i ~~						
y_s	-22.945	9.661	-2.375	0.018	-0.397	-0.397
m_i ~~						
m_s	-0.774	0.443	-1.749	0.08	-0.164	-0.164
y_i ~~						
m_i	0				0	0
m_s	0				0	0
y_s ~~						
m_i	0				0	0
m_s	0				0	0

Intercepts:

	Estimate	Std.Err	z-value	P(>|z|)	Std.lv	Std.all
impairment, week=0	0				0	0
impairment, week=2	0				0	0
impairment, week=4	0				0	0
impairment, week=6	0				0	0
impairment, week=8	0				0	0
insomnia, week=0	0				0	0
insomnia, week=2	0				0	0
insomnia, week=4	0				0	0
insomnia, week=6	0				0	0

insomnia, week=8	0				0	0
y_i	22.093	4.549	4.857	0	0.952	0.952
y_s	0.357	1.882	0.19	0.849	0.078	0.078
m_i	8.473	1.007	8.417	0	1.61	1.61
m_s	0.278	0.372	0.748	0.454	0.262	0.262

Variances:

	Estimate	Std.Err	z-value	P(>|z|)	Std.lv	Std.all
impairment, week=0	251.501	19.613	12.823	0	251.501	0.318
Impairment.1	251.501	19.613	12.823	0	251.501	0.332
Impairment.2	251.501	19.613	12.823	0	251.501	0.328
Impairment.3	251.501	19.613	12.823	0	251.501	0.308
Impairment.4	251.501	19.613	12.823	0	251.501	0.277
insomnia, week=0	5.183	0.325	15.962	0	5.183	0.158
Insomnia, week=1	5.183	0.325	15.962	0	5.183	0.162
insomnia, week=4	5.183	0.325	15.962	0	5.183	0.155
insomnia, week=6	5.183	0.325	15.962	0	5.183	0.139
insomnia, week=8	5.183	0.325	15.962	0	5.183	0.12
y_i	355.842	30.126	11.812	0	0.66	0.66
y_s	9.398	4.272	2.2	0.028	0.449	0.449
m_i	22.107	1.341	16.488	0	0.799	0.799
m_s	1.009	0.171	5.899	0	0.896	0.896

Growth Curve Mediation Model: Daytime Sleepiness and Activity Impairment

Latent Variables:

	Estimate	Std.Err	z-value	P(>|z|)	Std.lv	Std.all
y_i =~						
impairment, week=0	1				23.199	0.826
impairment, week=2	1				23.199	0.842
impairment, week=4	1				23.199	0.836
impairment, week=6	1				23.199	0.81
impairment, week=8	1				23.199	0.768
y_s =~						
impairment, week=0	0				0	0
impairment, week=2	1				4.513	0.164
impairment, week=4	2				9.026	0.325
impairment, week=6	3				13.539	0.473
impairment, week=8	4				18.052	0.598
m_i =~						
sleepiness, week=0	1				4.395	0.926
sleepiness, week=2	1				4.395	0.94
sleepiness, week=4	1				4.395	0.937
sleepiness, week=6	1				4.395	0.915
sleepiness, week=8	1				4.395	0.88
m_s =~						
sleepiness, week=0	0				0	0
sleepiness, week=2	1				0.653	0.14
sleepiness, week=4	2				1.307	0.278
sleepiness, week=6	3				1.96	0.408

sleepiness, week=8	4				2.613	0.523

Regressions:

	Estimate	Std.Err	z-value	P(>|z|)	Std.lv	Std.all
y_i ~						
female	4.588	1.696	2.705	0.007	0.198	0.099
hourly worker	9.374	1.749	5.361	0	0.404	0.197
racial minority	3.177	2.255	1.409	0.159	0.137	0.059
hispanic	0.576	2.803	0.206	0.837	0.025	0.009
chronic sleep dx	7.678	2.343	3.277	0.001	0.331	0.143
any chronic dx	-4.065	2.358	-1.724	0.085	-0.175	-0.088
chronic mh dx	17.865	2.625	6.806	0	0.77	0.359
y_s ~						
female	-0.405	0.894	-0.453	0.65	-0.09	-0.045
hourly worker	-1.275	1.101	-1.157	0.247	-0.282	-0.138
racial minority	1.053	1.106	0.952	0.341	0.233	0.1
hispanic	0.371	1.468	0.253	0.8	0.082	0.03
chronic sleep dx	-0.079	1.364	-0.058	0.954	-0.018	-0.008
any chronic dx	-0.621	1.173	-0.529	0.597	-0.138	-0.069
chronic mh dx	-0.888	1.185	-0.749	0.454	-0.197	-0.092
m_i ~						
female	0.222	0.327	0.679	0.497	0.051	0.025
hourly worker	0.787	0.338	2.328	0.02	0.179	0.087

racial minority	0.146	0.407	0.36	0.719	0.033	0.014
hispanic	0.706	0.474	1.489	0.136	0.161	0.058
chronic sleep dx	1.316	0.452	2.91	0.004	0.299	0.13
any chronic dx	-0.376	0.488	-0.771	0.44	-0.086	-0.043
chronic mh dx	0.444	0.516	0.861	0.389	0.101	0.047
m_s ~						
female	-0.067	0.102	-0.656	0.512	-0.102	-0.051
hourly worker	0.047	0.101	0.459	0.646	0.071	0.035
racial minority	-0.051	0.119	-0.427	0.669	-0.078	-0.033
hispanic	0.253	0.172	1.474	0.14	0.388	0.14
chronic sleep dx	0.114	0.122	0.936	0.349	0.174	0.075
any chronic dx	0.083	0.133	0.625	0.532	0.128	0.064
chronic mh dx	0.094	0.145	0.65	0.516	0.144	0.067
y_s ~						
group (c)	-1.263	2.114	-0.597	0.55	-0.28	-0.138
m_s ~						
group (a)	-0.257	0.094	-2.731	0.006	-0.393	-0.194
y_s ~						
m_s (b)	1.345	6.757	0.199	0.842	0.195	0.195

Covariances:

	Estimate	Std.Err	z-value	P(>|z|)	Std.lv	Std.all
y_i ~~						
y_s	-21.281	10.595	-2.009	0.045	-0.263	-0.263

m_i ~~						
m_s	-0.634	0.32	-1.978	0.048	-0.235	-0.235
y_i ~~						
m_i	0				0	0
m_s	0				0	0
y_s ~~						
m_i	0				0	0
m_s	0				0	0

Intercepts:

	Estimate	Std.Err	z-value	P(>|z|)	Std.lv	Std.all
impairment, week=0	0				0	0
impairment, week=2	0				0	0
impairment, week=4	0				0	0
impairment, week=6	0				0	0
impairment, week=8	0				0	0
sleepiness, week=0	0				0	0
sleepiness, week=2	0				0	0
sleepiness, week=4	0				0	0
sleepiness, week=6	0				0	0
sleepiness, week=8	0				0	0
y_i	22.172	4.409	5.028	0	0.956	0.956
y_s	1.602	2.11	0.759	0.448	0.355	0.355
m_i	6.677	0.91	7.341	0	1.52	1.52

m_s	-0.332	0.263	-1.26	0.208	-0.508	-0.508

Variances:

	Estimate	Std.Err	z-value	P(>|z|)	Std.lv	Std.all
impairment, week=0	251.467	19.243	13.068	0	251.467	0.318
impairment, week=2	251.467	19.243	13.068	0	251.467	0.331
impairment, week=4	251.467	19.243	13.068	0	251.467	0.327
impairment, week=6	251.467	19.243	13.068	0	251.467	0.307
impairment, week=8	251.467	19.243	13.068	0	251.467	0.276
sleepiness, week=0	3.216	0.295	10.886	0	3.216	0.143
sleepiness, week=2	3.216	0.295	10.886	0	3.216	0.147
sleepiness, week=4	3.216	0.295	10.886	0	3.216	0.146
sleepiness, week=6	3.216	0.295	10.886	0	3.216	0.14
sleepiness, week=8	3.216	0.295	10.886	0	3.216	0.129
y_i	354.738	29.43	12.053	0	0.659	0.659
y_s	18.399	6.599	2.788	0.005	0.903	0.903
m_i	18.371	1.158	15.864	0	0.951	0.951
m_s	0.396	0.089	4.466	0	0.927	0.927
